# Antagonism of epidermal growth factor receptor signaling favors hepatitis E virus life cycle

**DOI:** 10.1128/jvi.00580-24

**Published:** 2024-06-10

**Authors:** Kathrin Woytinek, Mirco Glitscher, Eberhard Hildt

**Affiliations:** 1 Division of Virology, Paul Ehrlich Institute, Langen, Germany; University of Southern California, Los Angeles, California, USA

**Keywords:** EGFR, erlotinib, multivesicular bodies, autophagy, innate immunity

## Abstract

Hepatitis E virus (HEV) poses a global threat, which currently remains understudied in terms of host interactions. Epidermal growth factor receptor (EGFR) plays multifaceted roles in viral pathogenesis, impacting host-cell entry, viral replication, and host-defense modulation. On the one hand, EGFR signaling emerged as a major driver in innate immunity; on the other hand, a crosstalk between HEV and EGFR requires deeper analysis. We therefore aimed to dissect the receptor’s involvement in the HEV life cycle. In persistently HEV-infected cells, the EGFR amount is decreased alongside with enhanced receptor internalization. As compared with the control ligand-induced EGFR, activation revealed an early receptor internalization and degradation in HEV-replicating cells, resulting in a notable EGFR signaling delay. Interestingly, inhibition or silencing of EGFR increased viral replication, extracellular and intracellular viral transcripts, and released infectious particles. The pro-viral impact of EGFR inhibition was attributed to (i) impaired expression of interferon-stimulated genes, (ii) activation of the autophagosomal system, (iii) virus-induced inhibition of lysosomal acidification, and (iv) a decrease of the cellular cholesterol level.

**IMPORTANCE:**

This study identifies epidermal growth factor receptor (EGFR) as a novel host factor affecting hepatitis E virus (HEV): EGFR downregulation promotes viral replication, release, and evasion from the innate immune response. The discovery that EGFR inhibition favors viral spread is particularly concerning for HEV patients undergoing EGFR inhibitor treatment.

## INTRODUCTION

The hepatitis E virus (HEV), a hepatotropic single-stranded RNA virus classified within the Paslahepevirus genus of the *Hepeviridae* family, has emerged as a significant global health concern (1, 2). Predominantly represented by genotypes 1–4 and 7, HEV’s human pathogenic forms present a spectrum of clinical manifestations, ranging from asymptomatic cases to chronic and fulminant hepatitis (3, 4). Immunocompromised patients and pregnant women, in particular, face a heightened risk of developing a severe disease course, with pregnancy-associated HEV infections showing a mortality rate of up to 30% (5–8). The primary mode of HEV transmission is through the fecal-oral route, typically resulting from the consumption of contaminated water or food (9). Moreover, in industrialized regions, there is a growing awareness of transfusion-related transmission (10–12). Despite its worldwide prevalence, with up to 20 million infections and 70,000 HEV-related deaths annually, there is no specific therapy available. Treatment options remain limited to ribavirin and pegylated interferon (13, 14). Treatment with interferon is associated with adverse effects affecting compliance, highlighting the urgent need for more effective solutions to combat this global health threat (15, 16). Improvement of our understanding of the viral life cycle and virus-host interaction might contribute to overcome this lack of adequate treatment and prevention options.

HEV release as quasi-enveloped HEV (eHEV) viral particles depends on multivesicular bodies (MVBs) (17–19). The viral pORF3 protein orchestrates a sophisticated interplay with cellular components, such as tumor susceptibility gene 101 and the endosomal sorting complex required for transport via its viral late-domain (PxxP motif) (20–22). The structural protein pORF3 interacts with the viral capsid protein pORF2 associated with genomic RNA, guiding it to the sides of progeny formation and facilitating invagination as intraluminal vesicles (23, 24). Recent findings indicate that HEV relies heavily on the tightly regulated homeostasis of the endosomal system, which is balancing between exosomal release and lysosomal degradation. In this context, modulation of intracellular cholesterol levels serves as a pivotal factor, directing the course of HEV within the cell (25). eHEVs are detected in cell culture supernatants and the bloodstream of infected patients. Upon reaching the biliary duct, these virions shed their membranous envelope, resulting in the secretion of naked HEV virions into the stool increasing infectivity up to 10-fold (18, 26).

The epidermal growth factor receptor (EGFR) is a tyrosine kinase receptor belonging to the ErbB family, governing essential processes such as cell proliferation, differentiation, division, and survival (27, 28). Activation of EGFR occurs through the binding of its ligand, for example, epidermal growth factor (EGF), to its extracellular ligand-binding domain (29). Ligand-induced dimerization leads to activation of EGFR intracellular tyrosine kinase domains and is thereby initiating downstream signaling cascades, including (i) the Ras-Raf-MEK-Erk, (ii) JAK-Stat, and (iii) PI3K-Akt-mTOR pathways (27, 29–31). To regulate initiation of signaling, the activated receptor undergoes clathrin-dependent endocytosis and subsequent degradation within the lysosomal compartment (32). With its involvement in the activation of multiple pathways, EGFR plays a critical role in cytoskeleton remodeling, regulation of the endosomal system, and modulation of innate immunity (33). Given the dependency of several viruses on these cellular processes, it is not surprising that various viruses interfere with EGFR to exploit it for their own benefit during the viral life cycle. Viral entry into host cells, induction of structural compartments necessary for viral replication, and inhibition of antiviral responses are among the key processes influenced by virus-dependent modulation of EGFR levels and functionality (34). The interplay between EGFR and notable viruses such as influenza A virus (IAV), hepatitis B virus (HBV), hepatitis C virus (HCV), zika virus (ZIKV), human cytomegalovirus (HCMV), and severe acute respiratory syndrome coronavirus 2 has already been studied (35–41). Notably, a recent report describes EGFR’s supportive role in the entry process of the hepatitis E virus without any impact on HEV genome replication or morphogenesis (42).

In light of the relevance of EGFR for the life cycle of a variety of viruses and its impact on innate immunity, this study specifically aims to investigate the intricate interplay between HEV and EGFR in order to gain new insights into the multifaceted effect of EGFR on HEV replication. In contrast to the abovementioned study by Schrader et al., a strong impact of EGFR modulation on HEV genome replication is observed. A deeper understanding of the EGFR-HEV crosstalk can contribute to the development of targeted therapeutic strategies against HEV infections.

## MATERIALS AND METHODS

### Cell culture and cell treatment

Within this study A549 cells, the highly HEV-susceptible subclone A549/D3, and A549/D3 cells infected with HEV genotype 3 strain KernowC1-p6 (A549/D3-HEV3p6) were cultivated. In addition, persistently HEV-infected A549 cells, parental A549 cells infected with HEV genotype 3c strain 47832c, were used (43, 44). The latter is a cell culture model of stable HEV infection gaining high HEV titers. The Chinese hamster ovary (CHO) cell line, lacking EGFR expression, was cultivated as control for EGFR silencing. For cell cultivation, Dulbecco’s modified Eagle’s medium (DMEM) was supplemented with 10% (vol/vol) fetal bovine serum (FBS.S 0615, Bio & Sell GmbH, Feucht, Germany), 100 U/mL penicillin, 100 µg/mL streptomycin, and 2 mM L-glutamine. The liver-derived Alexander cell line PLC-PRF-5 derived from a donor with hepatoma and PLC-PRF-5 cells persistently infected with HEV genotype 3c strain 47832c (PLC-PRF-5wt) were cultivated in minimum essential medium (MEM) supplemented with 10% (vol/vol) fetal bovine serum, 100 U/mL penicillin, 100 µg/mL streptomycin, 2 mM L-glutamine, and 1% non-essential amino acids. All cells were cultivated under the same conditions at 37°C with 95% relative humidity and 5% CO_2_ and passaged two times a week by trypsinization.

Treatment with 50 ng/mL epidermal growth factor (EGF) (Sigma-Aldrich, St. Louis, USA), 100 µM ribavirin (NV17217, Roche, Mannheim, Germany), 20 µM gefitinib (S1025, Selleckchem), and 12.5 µM or 25 µM EGFR inhibitor erlotinib (S7786; Selleckchem, Houston, USA) was performed in fully supplemented DMEM for A549-derived cell lines and fully supplemented MEM for PLC-PRF-5 cell lines. Inhibition of lysosomal or proteasomal protein degradation was performed using 200 µM leupeptin (A2183,0010, AppliChem, Darmstadt, Germany) or 100 nM bortezomib (S1013, Selleckchem) over 24 h. To interfere with lysosomal acidification, treatment with 25 nM bafilomycin A1 (BFLA, B1793, Sigma-Aldrich, St. Louis, USA) over 24 h was performed. Protein half-life assays were done using 100 mg/mL cycloheximide (CHX; Sigma-Aldrich, Hamburg, Germany) over 0 min to 180 min.

### Knockdown of EGFR with siRNA

Transfection with 10 nM siRNA targeting the EGFR (siEGFR, sc-29301, Santa Cruz Biotechnology, Dallas, USA) or control siRNA (scrambled control, Santa Cruz Biotechnology, Dallas, USA) was performed using the siPORT NeoFX Transfection Agent (AM4511, Thermo Fisher Scientific) according to the manufacturer’s overlay transfection instructions. Adjustments of the transfection protocol were made by using 6 µL of siPORT for 1.8 × 10^5^ cells per 6-well plate. A transfection mix containing siRNA and siPORT was incubated 15 min prior to overlay of cells. Knockdown was performed over 48 h and 72 h.

### RNA extraction, cDNA synthesis, and qRT-PCR

Isolation of total intracellular RNA was done using peqGOLD TriFast reagent (PEQLAB Biotechnologie GmbH, Erlangen, Germany) or RNA-Solv Reagent (Omega Bio-Tek, Norcross, USA) and chloroform as described by the manufacturer. Total RNA was digested with RQ1 DNase I (Promega, USA) and reverse transcribed using random hexamer primers and RevertAid H Minus Reverse Transcriptase (Thermo Fisher Scientific, USA). Analysis of gene expression was performed via quantitative reverse transcription-PCR (qRT-PCR) using the Maxima SYBR-Green qPCR Kit (Thermo Fisher Scientific, Braunschweig, Germany) and primers mentioned in Table 1. All procedures were performed according to the manufacturer’s instructions. Normalization of gene expression levels was carried out with measurement of the reference gene human ribosomal protein L27 (hRPL27) as previously described using the ΔΔC_T_ method (45, 46). Extracellular viral RNA in cell culture supernatant was quantified using the LightCycler Multiplex RNA Master Mix (Roche Diagnostics, Mannheim, Germany) and LightMix Modular Hepatitis E Virus Kit (TIB Molbio, Berlin, Germany) as described by Glitscher et al. (47). Analyses were performed with LightCycler 480 Instrument II (Roche, Mannheim, Germany).

**TABLE 1:** Primer sequences used for qRT-PCR analyses

Name of oligonucleotide	Sequence
Human-RPL27_fw	5′-AAA GCT GTC ATC GTG AAG AAC-3′
Human-RPL27_rev	5′-GCT GTC ACT TTG CGG GGG TAG-3′
HEV_ORF2_fw (subgenomic)	5′-GGT GGT TTC TGG GGT GAC-3′
HEV_ORF2_rev (subgenomic)	5′-AGG GGT TGG TTG GAT GAA-3′
HEV_ORF1-3_fw (genomic)	5′-TAC AGC GGG TGG AAT GAA TAA-3′
HEV_ORF1-3_rev (genomic)	5′-GCA GCA TAG GCA GAA GCA T-3′
Human-GBP1_fw	5′-GGT CCA GTT GCT GAA AGA GC-3′
Human-GBP1_rev	5′-TGA CAG GAA GGC TCT GGT CT-3′
OAS1_fw	5′-AGG TGG TAA AGG GTG GCT CC-3′
OAS1_rev	5′-ACA ACC AGG TCA GCG TCA GAT-3′
Tetherin_fw	5′-ACC TGC AAC CAC ACT GTG ATG-3′
Tetherin_rev	5′-CAA GCT CCT CCA CTT TCT TTT GTC-3′
ISG15_fw	5′-GAA CCT CTG AGC ATC CTG GTG AG-3′
ISG15_rev	5′-CAG AAC AGG TCG TCC TGC ACA C-3′
PKR_fw	5′-TTC ACT ACA ATG GCT GTT GGG ATG G-3′
PKR_rev	5′-TCA AAG AGT TCC AAA GCC AAA ACT TTG TCT AG-3′
MxA_fw	5′-CAG CAC CTG ATG GCC TAT CA-3′
MxA_rev	5′-ACG TCT GGA GCA TGA AGA ACT G-3′
STX17_fw	5′-GAG AAA TTG AGA AAC TTT GTT TG-3′
STX17_rev	5′-AAT GGA GTT GGA GAA ATT CTG C-3′
EGFR_fw	5′-TGG TTA TGT CCT CAT TGC-3′
EGFR_rev	5′-AGA TAA GAC TGC TAA GGC-3′
IFNα_fw	5′-TCT TCA GCA CAA AGG ACT CAT CTG CTG-3′
IFNα_rev	5′-CAT CAG GGG AGT CTC TGT CAC CC-3′
IFNβ_fw	5′-CAG GAT GAA CTT TGA CAT CCC TGA GG-3′
IFNβ_rev	5′-ACA ATA GTC TCA TTC CAG CCA GTG CTA G-3′

### Indirect immunofluorescence analyses

Cells were fixed with 100% ethanol (EtOH) for 30 min at −20°C or with 3.7% formaldehyde (FA) for 15 min at room temperature (RT). Permeabilization of FA-fixed cells was performed with 0.5% Triton X-100 in PBS for 10 min at RT. Afterward, cells were blocked for 15 min with 5% (wt/vol) bovine serum albumin (BSA) fraction V (Carl Roth, Karlsruhe, Germany) in TBS-T [Tris-buffered saline (20 mM Tris, 150 mM sodium chloride, pH 8.8), supplemented with 0.05% (vol/vol) Tween 20] in case of EtOH fixation and 5% BSA in TBS in case of FA fixation. Primary antibodies against pORF2 (HCD3K129, polyclonal rabbit α-pORF2, raised against aa112-608 of recombinant pORF2 protein), pORF3 (HPO3.1A6, purified polyclonal rabbit α-pORF3, raised against recombinant pORF3 protein), LAMP2 (polyclonal goat α-LAMP-2, AF6228; R&D Systems, Minneapolis, USA), EGFR (monoclonal mouse α-EGFR, sc-120, Santa Cruz Biotechnology), and CD63 (monoclonal mouse α-CD63, ab59479, Abcam) were incubated as previously described (47). Alexa Fluor 488/546/633 coupled donkey-α-rabbit/mouse/goat IgG (Invitrogen, Carlsbad, USA) served as secondary antibodies. Nuclei were stained with 4′,6-diamidino-2-phenylindole (DAPI, Carl Roth, Karlsruhe, Germany). Intracellular cholesterol staining was done using 200 µg/mL filipin III (F4767-1MG; Sigma-Aldrich, Schnelldorf, Germany) as described previously (48). One hour prior to 3.7% formaldehyde fixation, cells were cultured in cell culture medium supplemented with 75 nM LysoTracker Deep-Red (L12492, Thermo Fisher Scientific) at 37°C, 95% relative humidity, and 5% CO_2_. Stained cells were embedded on glass slides using Mowiol (Sigma-Aldrich, Hamburg, Germany). Imaging was performed with Leica SP8 confocal laser scanning microscopy (CLSM) (Leica, Wetzlar, Germany) using 100× magnification oil immersion objective (numerical aperture = 1.4), a scan speed of 200, image size of 2,048 × 2,048 pixels, and a pinhole set to 1.3 airy units. Z-stacks were performed with a step size of 0.25 µm. Subsequent processing was carried out with LASXcore software (Leica, Wetzlar, Germany). The generation of intensity profiles and calculation of thresholded Mander’s overlap coefficient (tMOC) or corrected total cell fluorescence (CTCF) were performed with FIJI software (49).

### SDS-PAGE and western blot analyses

The preparation of cell lysates for detection of proteins via sodium dodecyl sulfate polyacrylamide gel electrophoresis (SDS-PAGE) and western blot (WB) was carried out as described previously by Glitscher et al. (45). For the detection of phosphorylated proteins, cells were lysed with 200 µL of 1× cell lysis buffer (Cell Signaling Technology, Danvers, USA) freshly supplemented with Halt protease and phosphatase inhibitor cocktails (87785 and 78420, Thermo Fisher). Protein separation by SDS-PAGE was performed at 90 V to 120 V, followed by protein blotting on a polyvinylidene difluoride (Carl Roth, Karlsruhe, Germany) membrane using a semi-dry blotting method at 1.5 mA/cm^2^ gel (45, 50). If indicated, total protein stain was carried out directly after protein transfer using the Revert 700 Total Protein Stain Kit (Li-cor Biosciences GmbH, Bad Homburg, Germany) according to the manufacturer’s instructions. Ten percent milk in TBS-T or 1× RotiBlock (Carl Roth, Karlsruhe, Germany) was utilized to block membranes. Primary antibodies targeting rabbit α-pORF2 (1:3,000, previously described), rabbit α-EGFR (1:3,000, ab52894, Abcam), rabbit α-Erk (1:1,000, 4695S, Cell Signaling), rabbit α-phosphorylated EGFR (1:1,000, 2234S, Cell Signaling), mouse α-phosphorylated extracellular signal-regulated kinase (Erk) (1:1,000, 9106, Cell Signaling), guinea pig α-p62 (1:1,000, GP62-C, ProGen), rabbit α-MxA (1:1,000, HPA030917, Sigma Aldrich), goat α-Integrinβ1 (1:500, AF1778, R&D Systems), and rabbit α-PKR (1:1,000, 3072S, Cell Signaling) were incubated overnight at 4°C. Mouse α-GAPDH (1:10,000, sc-32233; Santa Cruz Biotechnology) or goat α-β-tubulin (1:2,000, ab21057, Abcam) served as reference protein. IRDye 680RD/IRDye 800CW-coupled antibodies (LI-COR Biosciences GmbH, Bad Homburg, Germany) were used as secondary antibodies, incubated for 1 h at room temperature. Signal detection was carried out with the LI-COR Odyssey CLx Imaging System (LI-COR Biosciences GmbH, Bad Homburg, Germany) and subsequent signal quantification via ImageStudio Lite software (v5.2; LI-COR Biosciences). Band signal intensities were normalized to the respective loading control (GAPDH, β-tubulin or total protein stain) and referred to its respective experimental control.

### Luciferase reporter assay

Stably electroporated A549/D3 cells containing a replicon construct harboring a Gaussia luciferase coding sequence inserted into the ORF2 region of HEV genotype 3 strain p6Kernow were treated with erlotinib (12.5 µM or 25 µM) 24 h after seeding. The HEV strain, kindly provided by Prof. Suzanne Emerson, and the stably electroporated A549/D3 cell line were described previously (51, 52). Cells were harvested after 24 h and 48 h erlotinib treatment using the Gaussia GLOW-Juice Kit (pjk, Kleinbittersdorf, Germany) according to the manufacturer’s protocol. An Orion II microplate luminometer (Titertek, Pforzheim, Germany) was used to measure the chemiluminescence resulting from the luciferase activity. Obtained values were normalized to the protein amount of the respective lysate determined via Bradford assay (Bradford reagent; Thermo-Scientific, Braunschweig, Germany). All methods were performed according to the manufacturer’s protocol.

### Determination of tissue culture infective dose

HEV-susceptible A549/D3 cells were seeded with a density of 0.25 × 10^4^ cells per 96-well plate. Twenty-four hours post seeding, cells were infected with 50 µL of cell culture supernatant collected from persistently HEV-infected A549 cells treated with erlotinib or gefitinib, transfected with EGFR siRNA, or respectively treated with DMSO or transfected with scrambled siRNA as control. A serial dilution with a ratio of 1:5 ranging from 1:5 to 1:78,125 was performed in six technical replicates. After 96 h of infection, cells were fixed with 100% EtOH for 1 h at −20°C with subsequent blocking in 5% (wt/vol) BSA (Carl Roth, Karlsruhe, Germany) diluted in TBS-T. The primary antibody against pORF2 (described previously) was incubated overnight at 4°C in blocking solution. After washing the cells with TBS-T, horseradish peroxidase-coupled donkey-α-rabbit IgG (NA934; GE Healthcare, Chicago, USA) was incubated for 1 h at room temperature in blocking solution. Staining of positive cells was performed directly after washing the cells with TBS-T using 3-amino-9-ethylcarbazol [staining solution: 30 mM Na-acetate, 12 mM acetic acid, 0.05% (wt/vol) 3-amino-9-ethylcarbazol, 0.01% H_2_O_2_]. Reaction was stopped with the addition of water. Tissue Culture Infective Dose (TCID_50_) values were calculated as previously described by evaluation of wells containing HEV-positive cells and presented as infectious units per mL (IU/mL) (53).

### FACS analysis

EGFR internalization upon EGF treatment was determined via fluorescence-activated cell sorting (FACS) analysis. Treated cells were harvested with Accutase (SCR005, Merck Millipore) at different time points post treatment. Fixation of cells was done for 20 min at 4°C with 4% formaldehyde if cells were not permeabilized or with the BD Cytofix/Cytoperm (554722, BD Biosciences) if permeabilization was necessary. Blocking was performed in 5% BSA diluted in FACS buffer (137 mM NaCl, 2.7 mM KCl, 8.1 mM Na_2_HPO_4_, 2% FCS, 1 mM EDTA, pH 7.4) for 10 min at room temperature. Primary antibodies against pORF2 (described previously) or EGFR (sc-120, Santa Cruz Biotechnology) were incubated 1 h at 4°C in FACS buffer for not permeabilized or (1×) BD Perm/Wash buffer (554723, BD Biosciences) for permeabilized cells. Cells were washed tree times with FACS buffer prior to 1 h incubation with secondary antibodies at 4°C. Donkey α-rabbit IgG coupled to Alexa Fluor 546 and donkey α-mouse IgG coupled to Alexa Fluor 488 (both Invitrogen, Carlsbad, USA) served as secondary antibodies. Fluorescence intensity was measured using a MACSQuant10 flow cytometer (Miltenyi) with 488-nm excitation and 525/50-nm (FITC channel) or 585/40-nm (PE channel) emission filters. Analyses were performed using FlowJo software (v10, FlowJo, LCC).

### Density gradient centrifugation

Preparation of discontinuous density gradients using iodixanol (OptiPrep; 1114542; Progen Biotechnik) and isopycnic centrifugation were performed as described previously (25). Cell culture supernatant of 48 h 25 µM erlotinib-treated persistently HEV-infected cells was analyzed. HEV RNA being present in the collected density fractions was analyzed using qRT-PCR. Each fraction was referred to the total amount of viral RNA present within the gradient to obtain a respective percentage.

### Profiling of protein kinases

Cells treated with 50 ng/mL EGF for 20 min and 2 h or with 25 µM erlotinib for 48 h were lysed with M-PER Mammalian Protein Extraction Reagent (Thermo Fisher Scientific) freshly supplemented with a Halt protease and phosphatase inhibitor cocktail (87785 and 78420, Thermo Fisher). Lysates were centrifuged for 15 min at 16,000 × *g* and 4°C. Obtained supernatants were centrifuged again for 15 min at 16,000 × *g* and 4°C. Samples were snap frozen as previously described (54). Protein quantification was performed using Pierce BCA Protein Assay Kit (23225, Thermo Fisher Scientific) according to the manufacturer’s instructions. Measurement was done using an Infinite M1000 Plate Reader (TECAN). Phosphotyrosine kinase (PTK) and serine/threonine kinase phosphorylation patterns (STK) were analyzed by a PamChip flow-through microarray assay on the PamStation 12 instrument (PamGene International). Determination of phosphorylation profiles and subsequent upstream kinase prediction via BioNavigator software were performed according to Alack et al. (54). Prediction for the differentially activated upstream kinases were performed by comparison of persistently HEV-infected cells to uninfected cells or additionally to their respective control group as described previously (55).

### Surface biotinylation

Surface proteins were biotinylated by incubation of cells with 0.5 mg/mL EZ-Link Sulfo-NHS-SS-Biotin (21331, Thermo Fisher Scientific) in PBS (137 mM NaCl, 2.7 mM KCl, 8.1 mM Na_2_HPO_4_, pH 7.4) supplemented with 1 mM MgCl_2_, 1 mM CaCl_2_ for 1 h at 4°C. Biotinylation solution was removed, and excess biotin was quenched by incubation of 50 mM NH_4_Cl in PBS with 1 mM MgCl_2_, 1 mM CaCl_2_ for 1 h at 4°C. Cells were lysed with RIPA buffer [50 mM Tris-HCl pH 7.2, 150 mM NaCl, 0.1% SDS (wt/vol), 1% sodium deoxycholate (wt/vol), 1% Triton X-100]. An aliquot of the cell lysate was prepared for western blot analyses (indicated as total) as described within the respective section. Biotinylated proteins were precipitated with Pierce NeutrAvidin Agarose Beads (29201, Thermo Fisher Scientific). Agarose beads were incubated for 2 h at 4°C with end-over-end mixing. After centrifugation at 2500 × *g* and 4°C for 2 min, supernatant was removed. Washing was performed four times by adding binding buffer consisting of PBS supplemented with 1% NP-40 and subsequent centrifugation for 2 min at 2,500 × *g* and 4°C. Biotinylated proteins were eluted by boiling the agarose beads for 15 min in 1× SDS-PAGE sample buffer, followed by centrifugation for 2 min at 2,500 × *g* and 4°C. The obtained supernatant was used for western blot analysis (indicated as surface).

### Entry assay

Infection of A549/D3 and PLC-PRF-5 cells with eHEV was performed for 1 h at 37°C, 95% relative humidity, and 5% CO_2_ with a multiplicity of infection (MOI) of 1.5. To determine compound-specific impacts on HEV entry, a 30-min pre-treatment with 25 µM erlotinib at 37°C, 95% relative humidity, and 5% CO_2_ was performed prior to infection. After infection, unbound and bound viruses were removed by washing four times with PBS and trypsinization. Detached cells were harvested and centrifuged 1 min at 5,000 × *g* to remove trypsin solution. Cell pellet was washed once again with PBS and centrifuged for 1 min at 5,000 × *g* and directly was used for RNA isolation with RNA-Solv Reagent (Omega Bio-Tek, Norcross, USA) and chloroform as described by the manufacturer. The internalized viral RNA amount was quantified using the LightCycler Multiplex RNA Master Mix (Roche Diagnostics, Mannheim, Germany) and LightMix Modular Hepatitis E Virus Kit (TIB Molbio, Berlin, Germany). Analyses were performed with LightCycler 480 Instrument II (Roche, Mannheim, Germany).

### Viability assay

Assays to determine the cytotoxicity of all compounds used during this study were performed in a 96-well plate. Cells treated with DMSO served as vehicle control, while the detergent Triton X-100 (1% vol/vol in growth medium) was used as internal control causing complete deterioration of cells. Subsequently, the redox-metabolic activity of viable cells was determined by utilizing the PrestoBlue Cell Viability Reagent (Thermo‐Scientific, Braunschweig, Germany) according to the manufacturer’s instructions. NADH/H+‐dependently formed Resorufin was measured with a plate reader (Infinite M1000; TECAN, Switzerland) at Ex560/Em590. Redox metabolic activity in treated cells was referred to the vehicle control.

### Statistical analysis

Data shown within this study are based on a minimum of three independent biological experiments (N), unless stated otherwise. Results are displayed as mean ± standard errors of the means. Data sets presented as fold change were referred to their respective experimental control group. This analysis was performed for each of the independent experiments. As the control groups were arbitrarily set as 1 or 100%, standard errors of the mean for these groups cannot be shown. Details regarding the conducted technical replicates (*n*) are provided within the figure legends. Calculation of statistical significance was determined using unpaired *t*-tests with Holm-Sidak correction for multiple-group comparison with GraphPad Prism 9.5 (GraphPad Software, USA). *P* values below 0.05 were considered as significant. Outliers were identified by performing the ROUT method (*Q* = 1%).

## RESULTS

### The EGFR amount is significantly reduced in persistently HEV-replicating cells

**Fig 1:**
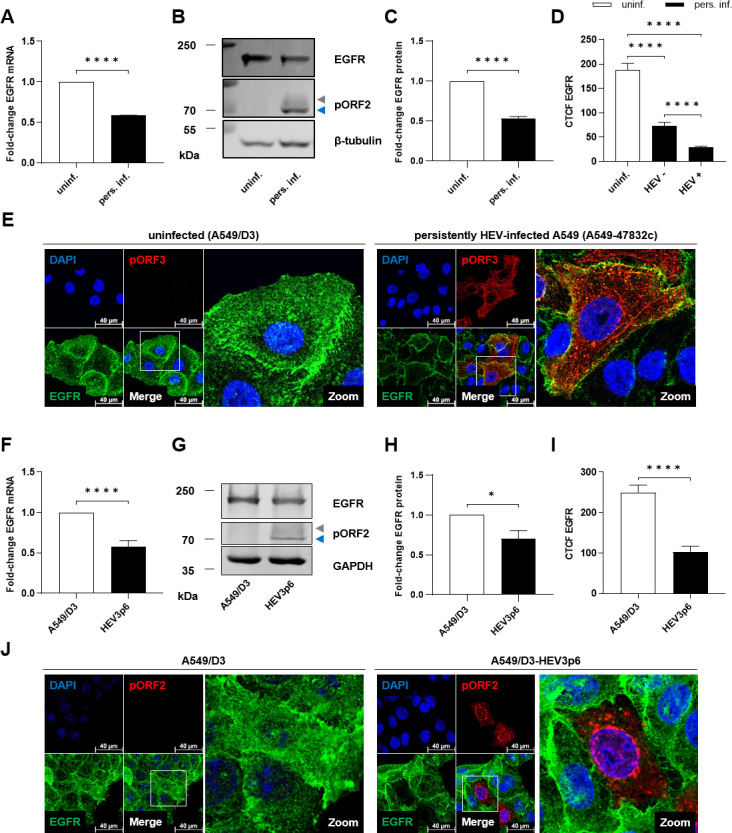
Significantly reduced EGFR amount in persistently HEV-replicating cells. (**A and F**) Relative change in intracellular EGFR mRNA amount measured via qRT-PCR. Values of persistently HEV-infected A549 cells infected either with HEV3 47832c (pers. inf.) (**A**) or KernowC1-p6 strain (A549/D3-HEV3p6) (**F**) were referred to uninfected controls (A549/D3) after normalization toward the respective reference gene human ribosomal protein L27 (hRPL27). Data of *N* = 3 (**A**) or *N* = 7 (**F**) independent experiments are presented as mean with SEM. (**B and G**) Representative western blot of EGFR and pORF2 (blue = unglycosylated, gray = glycosylated) in lysates of uninfected A549/D3 (uninf.) and either HEV3 47832c- (pers. inf.) (**B**) or KernowC1-p6 strain- (A549/D3-HEV3p6) (**G**) infected A549 cells. β-Tubulin or GAPDH were detected as internal loading controls. (**C and H**) Quantification of the relative amount of EGFR in panel B (**C**) or panel G (**H**). Values of *N* = 3 independent experiments are normalized to β-tubulin or GAPDH and referred to the respective uninfected control. (**D**) Quantification of EGFR intensity in confocal microscopy images shown in panel E depicted as CTCF per cell in uninfected (uninf.) and persistently HEV-infected A549 (pers. inf.) cells being pORF3 negative (HEV−) or pORF3 positive (HEV+); *N* = 2; *n* ≥ 37. (**E**) Representative confocal microscopy images of uninfected (left) and persistently HEV-infected (right) A549 cells immunostained for pORF3 (red) and EGFR (green) after fixation with EtOH. Nuclei were counterstained with DAPI (blue). Cell shown in the zoom section is indicated within the merge image. Scale bar: 40 µm. (**I**) CTCF quantification of EGFR intensity in confocal microscopy images shown in panel J; *n* ≥ 18. (**J**) Representative confocal microscopy images of uninfected (left) and persistently KernowC1-p6-infected A549/D3 cells (A549/D3-HEV3p6) (right) immunostained for pORF2 (red) and EGFR (green) after EtOH fixation. Nuclei were counterstained with DAPI (blue). Cell shown in the zoom section is indicated within the merge image. Scale bar: 40 µm. Microscopy was performed on Leica TCS SP8 system with 100× objective (numerical aperture 1.4). Statistical analysis was performed using unpaired *t*-tests with Holm-Sidak correction for multiple-group comparison related to uninfected control cells: *P < 0.05 and ****P < 0.0001.

To study the impact of an HEV infection on EGFR, the host protein was analyzed in persistently HEV-infected A549 cells. The analyses included two distinct HEV genotype 3 strains: the 47832c strain and the KernowC1 p6 strain. Here, mRNA levels, protein amount, and subcellular localization were assessed via qRT-PCR, WB, and CLSM, respectively (Fig. 1). Persistent HEV infection led to a notable decrease in EGFR mRNA compared with the uninfected control (Fig. 1A and F). In line with this, the reduced level of EGFR was reflected by a decreased protein amount as evidenced by western blot analyses (Fig. 1B, C, G and H). This reduction in EGFR was validated via CLSM on the single-cell level (Fig. 1D, E, I and J). Interestingly, the level of EGFR was slightly reduced even in pORF3-negative cells within the population of persistently infected A549 cells compared with uninfected controls, but the reduction was much less pronounced as compared with pORF3-positive cells (Fig. 1D). Importantly, EGFR displayed a dominant localization to the plasma membrane in HEV-infected cells, which stands in contrast to the more cellular distribution in uninfected cells.

These data indicate that persistent HEV infection leads to a downregulation of EGFR mRNA and protein level and affects the intracellular distribution of EGFR. Importantly, this effect was observed for two different HEV strains, underscoring a consistent impact on EGFR across different strains of viral genotype 3.

### Reversal of HEV-induced effects on the EGFR level by ribavirin treatment

**Fig 2:**
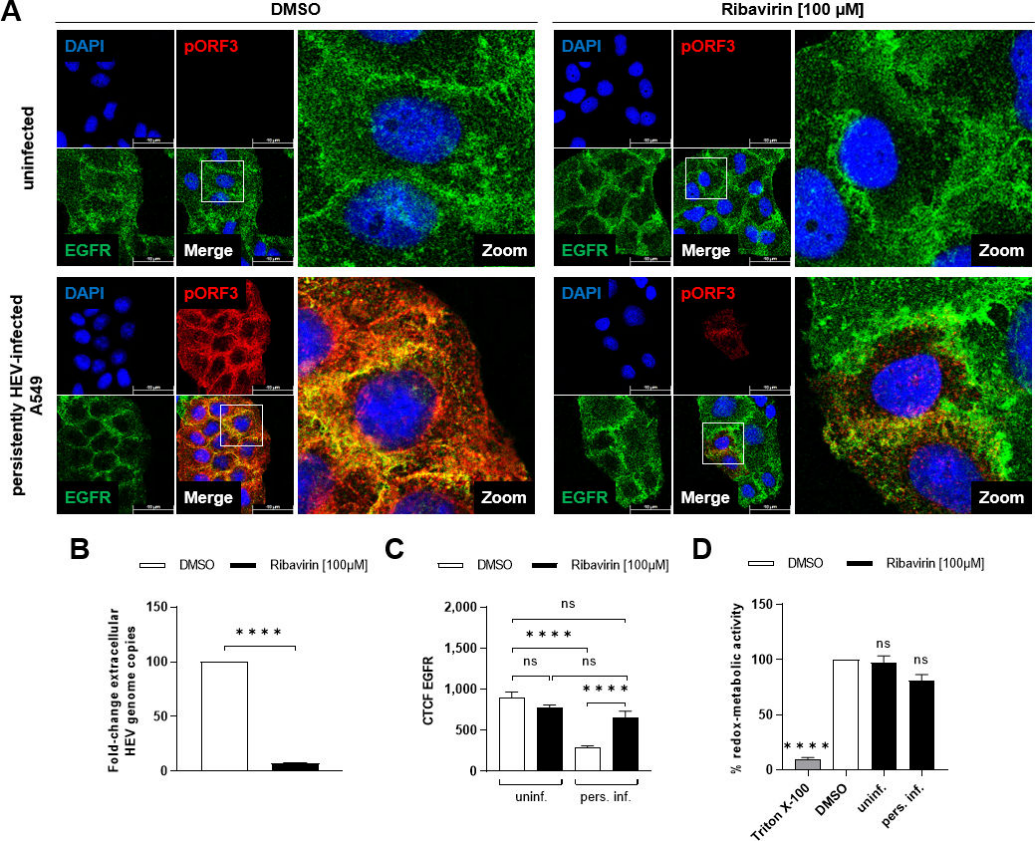
Ribavirin treatment reverses the HEV-induced decrease in EGFR level. (**A**) Representative confocal microscopy images depicting 48 h control-treated (DMSO) or ribavirin (100 µM)-treated uninfected and persistently HEV-infected A549 cells, immunostained for pORF3 (red) and EGFR (green) after EtOH fixation. Nuclei were counterstained with DAPI (blue). Cell shown in the zoom section is indicated within the merge image. Scale bar: 40 µm. (**B**) Fold change of extracellular HEV RNA from persistently HEV-infected A549 cells shown in panel A by qRT-PCR; unpaired *t*-test: ****P < 0.0001. (**C**) CTCF quantification of EGFR intensity in confocal microscopy images shown in panel A; *N* = 2; *n* ≥ 13. (**D**) Relative metabolic activity of uninfected and persistently HEV-infected A549 cells treated with ribavirin (100 µM) for 48 h assessed with a PrestoBlue cell viability assay; *N* = 3. DMSO-treated and 1% Triton X-100-treated cells served as negative and positive controls. Statistical analysis was performed using unpaired *t*-tests with Holm-Sidak correction for multiple-group comparison: ns: not significant; ****P < 0.0001.

**Fig 3:**
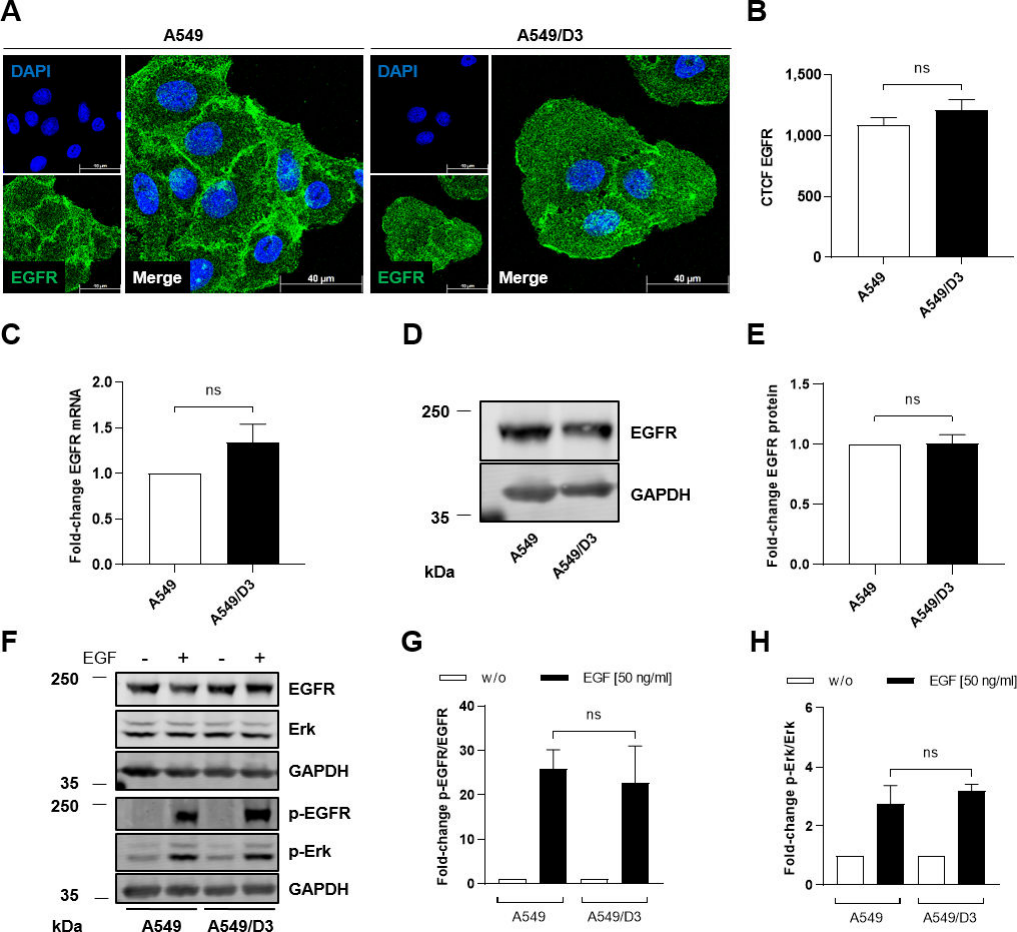
No difference in EGFR expression and EGF-induced phosphorylation between HEV-susceptible subclone A549/D3 and parental cell line A549. (**A**) Representative confocal microscopy images of A549/D3 and A549 cells immunostained for EGFR (green) after EtOH fixation. Nuclei were counterstained with DAPI (blue). Scale bar: 40  µm. (**B**) CTCF quantification of EGFR intensity in confocal microscopy images shown in panel A; *N* = 2; *n* = 16. (**C**) Relative change in intracellular EGFR mRNA amount in A549/D3 cells as compared with A549 cells measured via qRT-PCR. Each value was normalized toward the respective reference gene hRPL27; *N* = 3. (**D**) Representative western blot of EGFR protein amount in lysates of A549/D3 and A549 cells. GAPDH was detected as internal loading control. (**E**) Quantification of the relative amount of EGFR in A549/D3 as compared with A549 cells in panel D; *N* = 3. Values were normalized to GAPDH. (**F**) Representative western blot of total EGFR, total Erk, phosphorylated EGFR (p-EGFR), and phosphorylated Erk (p-Erk) in lysates of A549/D3 and A549 cells stimulated with EGF (50 ng/mL) for 30 min. GAPDH was detected as loading control. (**G**) Quantification of the relative phosphorylated EGFR protein amount (p-EGFR/total EGFR) in EGF-treated A549/D3 and A549 cells compared with the respective untreated control shown in panel F. Values are normalized to the respective GAPDH amount; *N* = 3. (**H**) Quantification of the relative phosphorylated Erk protein amount (p-Erk/total Erk) in EGF-treated A549/D3 and A549 cells compared with the respective untreated control shown in panel F. Values are normalized to the respective GAPDH amount; *N* = 3. Statistical analysis was performed using unpaired *t*-test comparing A549/D3 cells with A549 cells as indicated: ns: not significant.

To further corroborate the specific impact of HEV on EGFR subcellular distribution and amount, stable HEV-replicating cells were cured by ribavirin treatment and the effect on EGFR was studied (Fig. 2). Ribavirin exhibits potent antiviral effects on HEV, resulting in a notable reduction in viral genomes in the supernatant (Fig. 2B). The antiviral treatment leads to the restoration of EGFR levels in prior HEV-infected cells to those observed in uninfected cells (Fig. 2A and C), without showing any cytotoxicity (Fig. 2D). Importantly, the highly HEV-permissive subclone A549/D3 serves as a suitable control for parental A549 cells, displaying no differences in EGFR cellular distribution (Fig. 3A), mRNA level (Fig. 3C), or protein level (Fig. 3B, D and E). Even its response to EGF stimulation regarding the phosphorylation level of the receptor and its downstream target, Erk, showed no altered behavior as compared with A549 cells (Fig. 3F through H). In conclusion, the compensatory impact of ribavirin on the EGFR level, achieved by cure of HEV-infected cells, confirms HEV’s role in modulating the EGFR level.

### Augmented level of internalized EGFR in persistently HEV-infected cells

**Fig 4:**
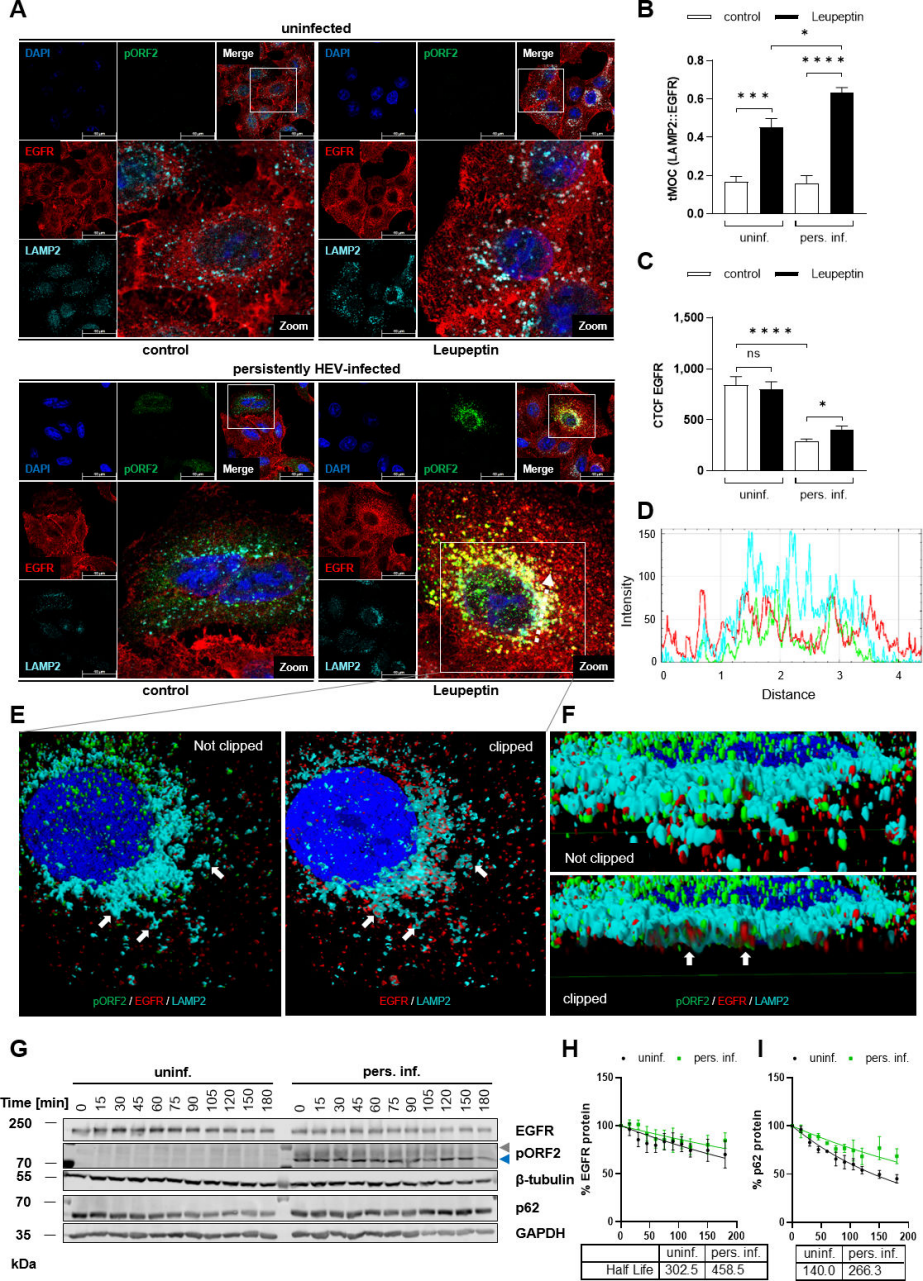
HEV-replicating cells show increased internalization of EGFR into lysosomal structures. Uninfected and persistently HEV-infected A549 cells were treated with 200 µM leupeptin for 24 h. (**A**) Representative confocal microscopy images of uninfected (top) and persistently HEV-infected (bottom) A549 cells immunostained for pORF2 (green), EGFR (red), and LAMP2 (cyan) after EtOH fixation. Nuclei were counterstained with DAPI (blue). Cell shown in the zoom section is indicated within the merge image. Scale bar: 40 µm. (**B**) Quantification of co-localization between cyan LAMP2 signal and red EGFR signal in panel A, depicted as tMOC per cell; *N* = 2; *n* ≥ 10. Data are presented as mean with SEM. (**C**) CTCF quantification of EGFR intensity in confocal microscopy images described in panel A; *n* = 20. Statistical analysis was performed using unpaired *t*-tests with Holm-Sidak correction for multiple-group comparison: ns: not significant; *P < 0.05, ***P < 0.001, and ****P < 0.0001. (**D**) Profile plot of region indicated in panel A (white arrow) corroborates the localization of EGFR (red) and HEV pORF2 (green) in LAMP2-positive structures (cyan). (**E and F**) 3D reconstruction of cell depicted in panel A clipped within the z-axis (**E**) or y-axis (**F**). White arrows indicate EGFR (red) inside LAMP2-positive structures (cyan). (**G**) Determination of EGFR protein half-life via a CHX chase assay in uninfected (uninf.) and persistently HEV-infected A549 cells (pers. inf.). Representative western blot of HEV pORF2 (blue = unglycosylated, gray = glycosylated), EGFR, p62, β-tubulin, and GAPDH over a time of 3 h post CHX treatment. (**H and I**) Quantification of EGFR (**H**) or p62 (**I**) amounts in panel G, shown as relative change in protein amount referred to 0 min treatment (set to 100%); *N* ≥ 3. A one-phase decay model was applied with an intercept of *y*(0) = 100 and a plateau of *y* = 0. Microscopy was performed on Leica TCS SP8 system with 100× objective (numerical aperture 1.4).

The aforementioned data pointed toward a reduced amount of EGFR in persistently HEV-infected cells. EGFR levels are intricately regulated by receptor internalization, recycling, and degradation. To investigate whether alterations in EGFR internalization contribute to the observed reduction of EGFR levels in persistent HEV infection, lysosomal degradation of EGFR was blocked by leupeptin. Immunofluorescence analyses of EGFR and lysosomal-associated membrane protein 2 (LAMP2) revealed an augmented intracellular co-localization between EGFR- and LAMP2-positive structures upon leupeptin treatment in HEV-positive cells. Notably, this was not the case in the absence of leupeptin, showing that differences in EGFR internalization only become apparent when the degradative process was impaired (Fig. 4A and B). Quantification of the co-localization confirmed this finding (Fig. 4B). A signal profile plot further corroborated these data about the overlap between EGFR and LAMP2 in leupeptin-treated HEV-positive cells (Fig. 4D). Subsequent z-stack reconstruction illustrated the localization of EGFR in the interior of LAMP2-positive structures (Fig. 4E and F). The elevated co-localization of EGFR with lysosomal structures following leupeptin treatment in HEV-infected cells corresponded with a notable increase in EGFR level (Fig. 4C). Nonetheless, this EGFR level did not reach the level of uninfected controls.

Considering the pronounced internalization of EGFR into lysosomal structures in HEV-replicating cells, coupled with the rise of the EGFR level upon inhibition of lysosomal function in HEV-infected cells, we assessed EGFR protein half-life by western blot analyses following a CHX chase assay. Here, no significant impact of persistent HEV infection on EGFR half-life as compared with the respective uninfected control was observed (Fig. 4G and H). As a control, the ubiquitin-binding protein p62, highly reliant on autolysosomal degradation, exhibited a significant increase in protein half-life in persistently HEV-infected cells (Fig. 4I).

In summary, these findings indicate an HEV-dependent impact on EGFR internalization, characterized by increased localization of EGFR toward LAMP2-positive structures. Despite the observed increase in receptor internalization, no significant reduction in EGFR half-life was detected.

### HEV-infection enhances EGFR internalization kinetics upon EGF stimulation

The functionality of EGFR as a receptor is highly dependent on its localization on the plasma membrane, which can be regulated by processes such as receptor internalization, recycling, or lysosomal degradation following prolonged stimulation with its natural ligands (56–58). Given the observed increase in EGFR internalization in HEV-positive cells, we investigated whether HEV-replicating cells and uninfected cells differ with respect to the ratio of surface EGFR to the total EGFR protein amount. Surface biotinylation and subsequent pulldown of biotinylated proteins were conducted for this purpose.

**Fig 5:**
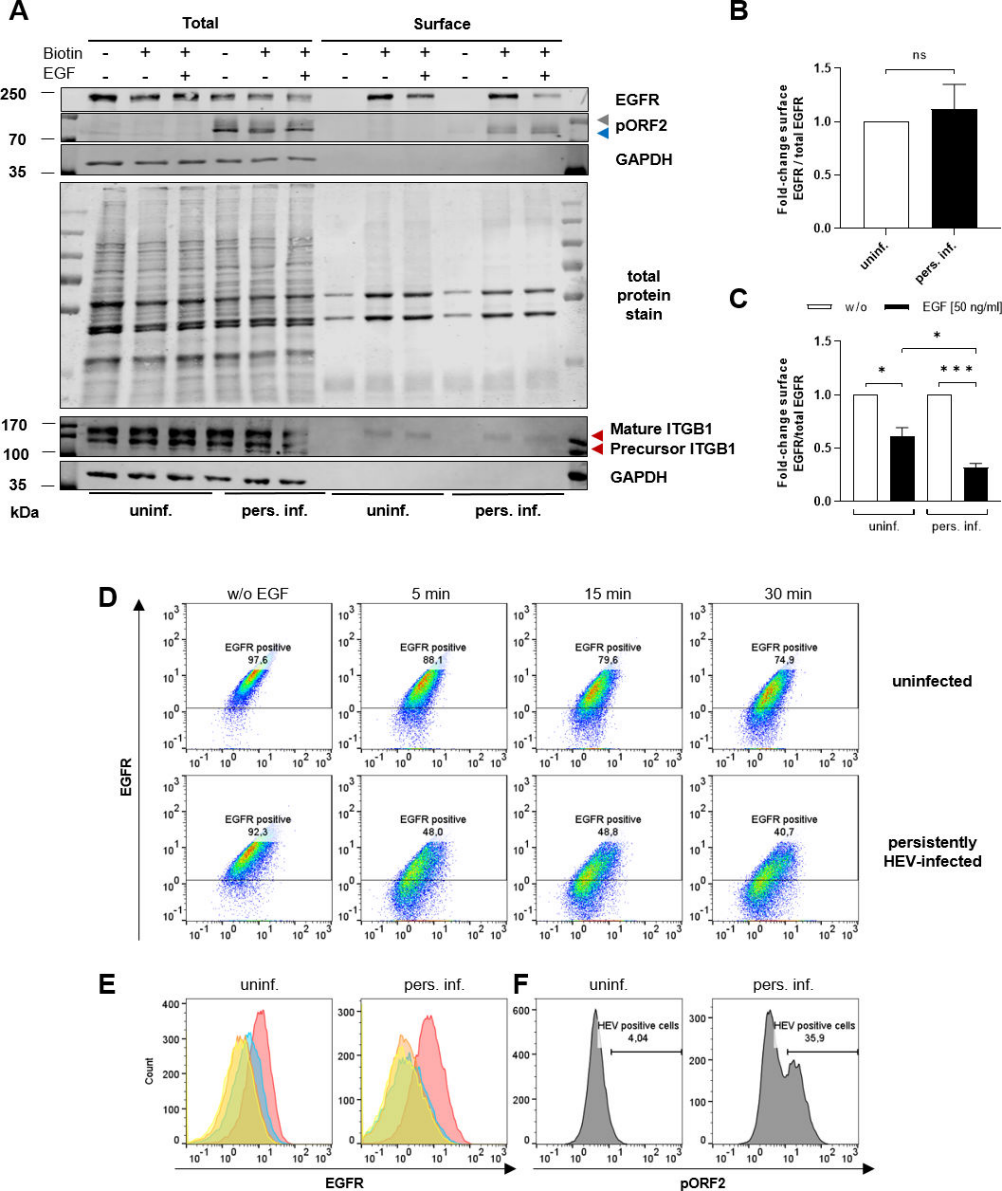
EGFR internalization upon EGF treatment occurs earlier in persistently HEV-infected cells. Surface proteins of uninfected and persistently HEV-infected A549 cells were biotinylated and precipitated using NeutrAvidin-agarose beads-based pulldown. Internalization of EGFR was induced with 50 ng/mL EGF for 30 min. (**A**) Representative western blot of EGFR, HEVpORF2 (blue = unglycosylated, gray = glycosylated), integrin beta-1 (ITGB1), and GAPDH in lysates of uninfected and persistently HEV-infected A549 cells before (total) and after pulldown (surface). A total protein stain was detected as loading control. (**B**) Quantification of the relative amount of surface EGFR in untreated (w/o EGF) persistently HEV-infected A549 cells as compared with the respective uninfected control shown in panel A. Surface EGFR/total EGFR amount was normalized to respective total protein amount; *N* = 3; unpaired *t*-test: ns: not significant. (**C**) Quantification of the relative amount of surface EGFR in EGF-treated uninfected or persistently HEV-infected A549 cells as compared with the respective untreated control shown in panel A. Surface EGFR/total EGFR amount was normalized to respective total protein amount; *N* = 3. Statistical analysis was performed using unpaired *t*-tests with Holm-Sidak correction for multiple-group comparison: ns: not significant; *P < 0.05 and ***P < 0.001. (**D and E**) Flow cytometry analysis of surface EGFR in uninfected and persistently HEV-infected A549 cells treated with 50 ng/mL EGF for 5 min, 15 min, and 30 min. Cell fixation was performed by FA under non-permeabilized conditions. Surface EGFR in the FITC channel displayed in representative dot blots, with indicated percentage of surface EGFR-positive cells (**D**), as well as in representative histograms (**E**). Histogram overlay: red = w/o EGF, blue = 5 min, orange = 15 min, and yellow = 30 min. (**F**) Flow cytometry analysis of HEV pORF2 (PE channel) in uninfected and persistently HEV-infected A549 cells shown in representative histograms. Percentage of HEV-positive cells are indicated within panel F. Cell fixation was performed by EtOH under permeabilized conditions.

The EGFR surface amounts did not differ between uninfected and HEV-infected cells (Fig. 5A and B). However, data described above indicate a reduced total EGFR protein amount in HEV-replicating cells, which in turn is relevant for receptor functionality. As EGFR signaling is modulated by stimulation-dependent receptor internalization, EGFR was stimulated with EGF and receptor uptake was determined in the presence or absence of HEV.

Interestingly, EGF treatment reduced the EGFR surface amount to a greater extent in HEV-infected cells as compared with the uninfected control (Fig. 5C). Therefore, further investigation of the kinetics of EGFR internalization induced by EGF treatment was performed. FACS analyses of surface EGFR levels confirmed comparable numbers for untreated cells either uninfected or HEV infected (Fig. 5D). However, over a 30-min period of EGF treatment, a significant and earlier decrease in the amount of surface EGFR was evident for HEV-persistent cells, with approximately half the amount detected as early as 5 min after treatment (Fig. 5D through F). While the surface EGFR levels in uninfected cells declined gradually over time upon EGF treatment, HEV-infected cells exhibited an immediate and sustained reduction, continuing to decrease after 30 min of treatment.

These data indicate that, although the surface EGFR amounts are comparable between uninfected and HEV-persistent cells, the internalization of EGFR following EGF stimulation occurs significantly earlier in infected cells as compared with the respective uninfected control.

### Accelerated degradation of EGFR following activation in persistently HEV-infected cells

**Fig 6:**
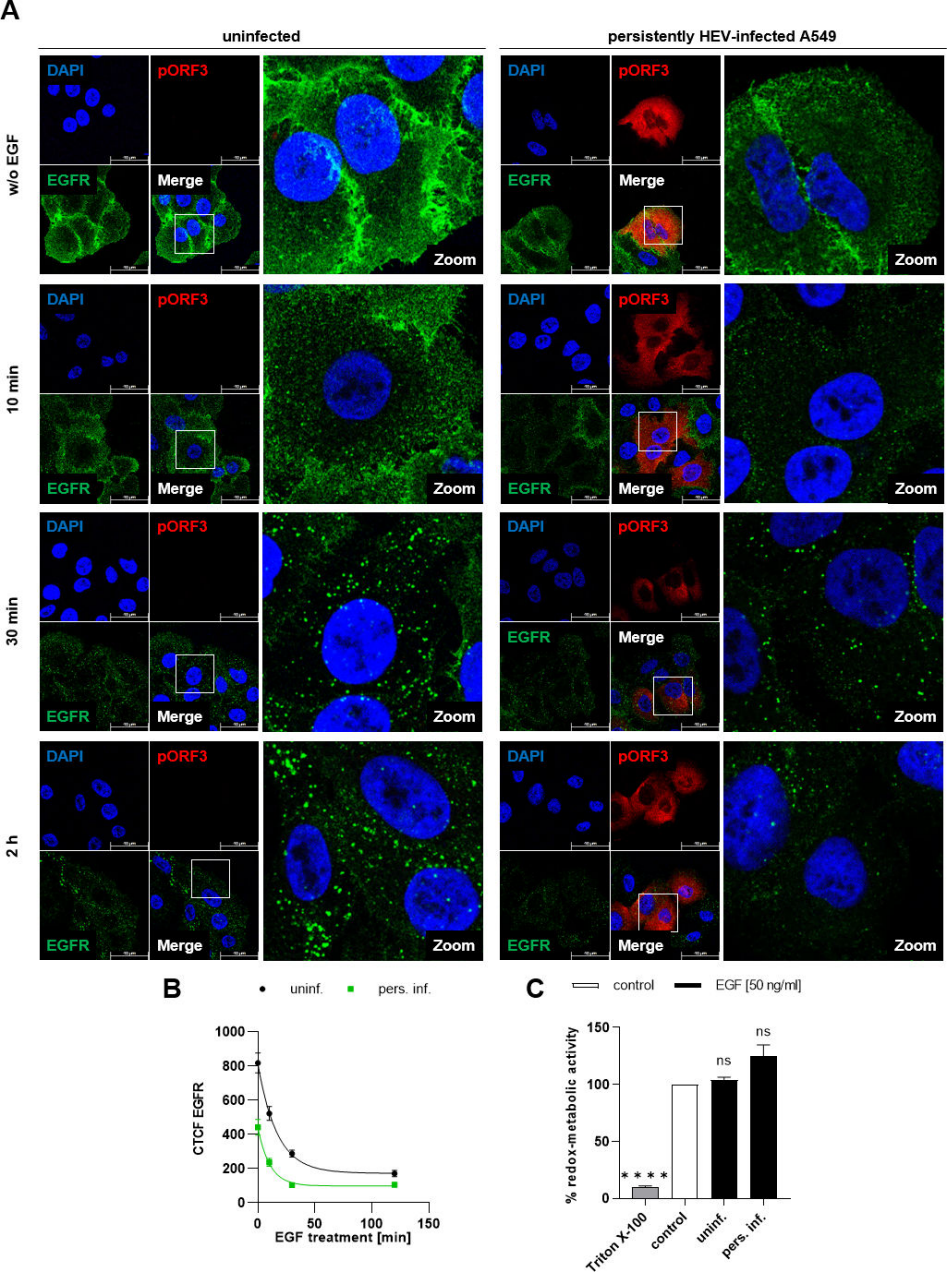
The accelerated internalization of EGFR induced by EGF in HEV-infected cells is associated with an enhanced degradation. (**A**) Representative confocal microscopy images of EGF-treated (50 ng/mL) uninfected and persistently HEV-infected A549 cells immunostained for pORF3 (red) and EGFR (green) after FA fixation. Nuclei were counterstained with DAPI (blue). EGF treatment was performed for 10 min, 30 min, and 2 h. Cell shown in the zoom section is indicated within the merge image. Scale bar: 40 µm. (**B**) CTCF quantification of EGFR intensity in confocal microscopy images shown in panel A; *N* = 2; *n* ≥ 14. A one-phase decay model was applied. (**C**) Relative metabolic activity of uninfected and persistently HEV-infected A549 cells treated with EGF (50 ng/mL) for 48 h assessed with a PrestoBlue cell viability assay; *N* = 3. Statistical analysis was performed using unpaired *t*-tests with Holm-Sidak correction for multiple-group comparison: ns: not significant; ****P < 0.0001.

**Fig 7:**
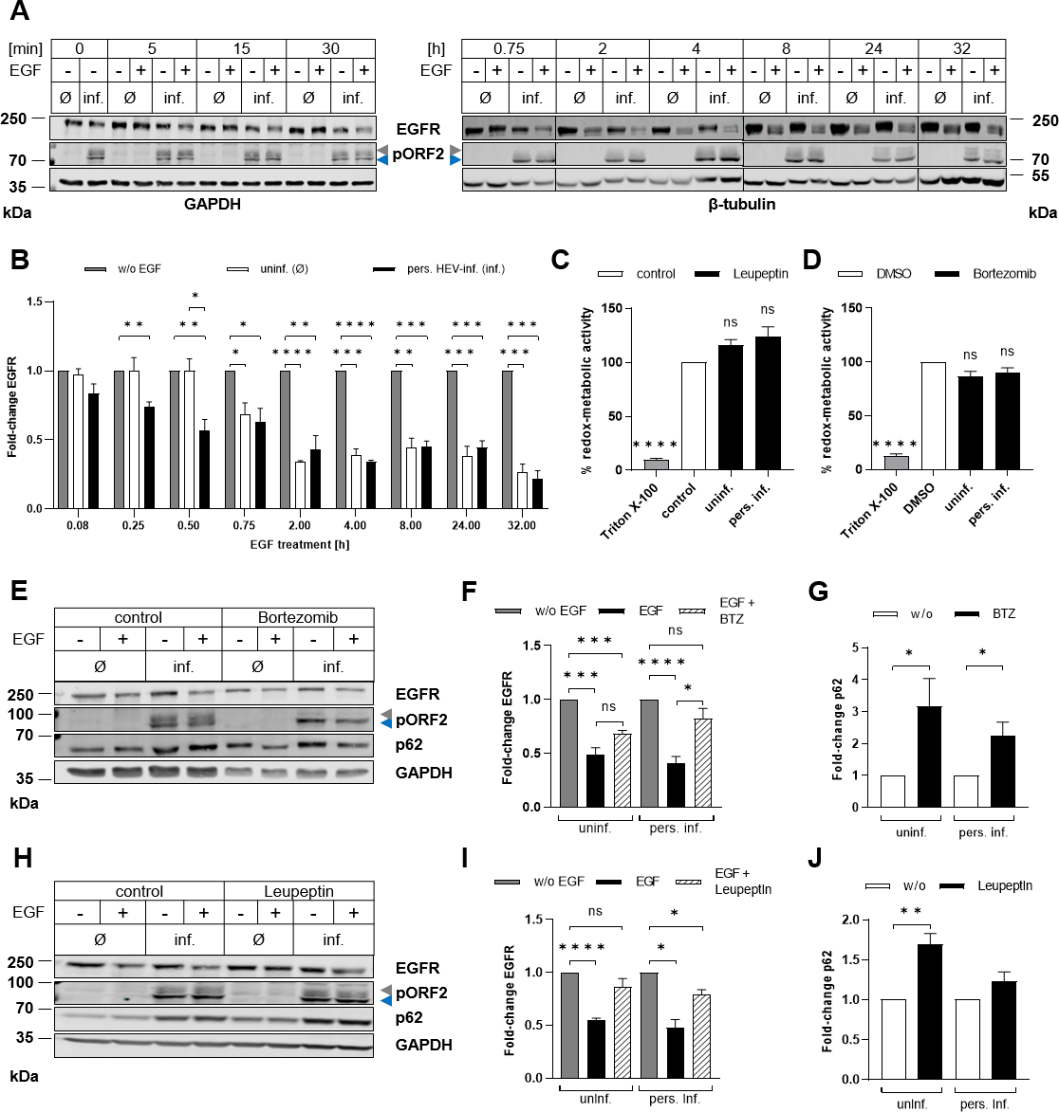
The degradation of EGFR following EGF treatment in HEV-replicating cells depends on the proteasomal degradation pathway. Uninfected and persistently HEV-infected A549 cells were treated with 50 ng/mL EGF for up to 32 h and analyzed for EGFR protein amount. (A) Representative western blot of EGFR and HEV pORF2 (blue = unglycosylated, gray = glycosylated) in lysates of uninfected and persistently HEV-infected A549 cells treated with EGF. GAPDH and β-tubulin were detected as loading control. (B) Quantification of the relative EGFR protein amount in EGF-treated uninfected and persistently HEV-infected A549 cells as compared with the respective untreated control shown in panel A; *N* = 3 independent experiments are shown. Values are normalized to the respective loading control. (C and D) Relative metabolic activity of uninfected and persistently HEV-infected A549 cells treated with leupeptin (200 µM) (C) or bortezomib (100 nM) (D) for 24 h assessed by a PrestoBlue cell viability assay; *N* = 3. (E and H) Representative western blot of EGFR, HEV pORF2, and p62 in lysates of uninfected and persistently HEV-infected A549 cells, which were treated for 24 h with bortezomib (BTZ) (100 nM) (E) or leupeptin (200 µM) (H) prior to 2 h 50 ng/mL EGF. GAPDH was detected as loading control. (F and I) Quantification of the relative EGFR protein amount in EGF-treated uninfected and persistently HEV-infected A549 cells as compared with the respective untreated (w/o EGF) control shown in panel E (F) or panel H (I); *N* = 3. Values are normalized to the respective GAPDH amount. Statistical analysis was performed to the respective untreated cells (w/o EGF) using unpaired *t*-tests with Holm-Sidak correction for multiple-group comparison. (G) Quantification of the relative p62 protein amount in BTZ-treated uninfected and persistently HEV-infected A549 cells as compared with the respective untreated (w/o BTZ, w/o EGF) control shown in panel E; *N* = 3. Values are normalized to the respective GAPDH amount. (J) Quantification of the relative p62 protein amount in leupeptin-treated (w/o EGF) uninfected and persistently HEV-infected A549 cells as compared with the respective untreated (w/o leupeptin, w/o EGF) control shown in panel H; *N* = 3. Values are normalized to the respective GAPDH amount. Statistical analysis was related to the respective untreated control (w/o leupeptin or BTZ) using unpaired *t*-tests with Holm-Sidak correction for multiple-group comparison: ns: not significant; *P < 0.05, **P < 0.01, ***P < 0.001, and ****P < 0.0001.

As demonstrated above, EGFR internalization upon EGF stimulation occurs rapidly in HEV-replicating cells. This raised the question whether HEV influences receptor degradation rates, thereby affecting signaling capacity. We subjected persistently HEV-infected cells and uninfected controls to EGF treatment for up to 2 h and assessed the EGFR amount using CLSM analysis (Fig. 6). EGF stimulation induced the internalization of EGFR in both cell types, irrespective of infection (Fig. 6A). Interestingly, HEV-infected cells exhibited a rapid reduction in EGFR level following EGF treatment, characterized by a higher rate constant of 0.096 min^−1^ (*R*^2^ = 0.63) compared with 0.06 min^−1^ (*R*^2^ = 0.73) observed in uninfected cells (Fig. 6B). Here, EGF did not exhibit any cytotoxic effects (Fig. 6C). To investigate differences in EGFR degradation following EGF treatment between HEV-replicating and uninfected cells, both cell lines were subjected to EGF treatment for up to 32 h. Samples were collected at various time points and analyzed by western blot analyses (Fig. 7A and B).

Here, a time-dependent decrease in total EGFR levels upon treatment was observed, which was independent from HEV infection (Fig. 7B). However, in persistently HEV-infected cells, EGFR degradation already occurred at earlier time points as compared with uninfected cells. This is in accordance with previous observations of accelerated receptor internalization. In HEV-infected cells, EGFR degradation was evident as early as 10 min after EGF stimulation, which stands in contrast to 45 min in uninfected cells. This enhancement is leveled out at 2 h post stimulation.

To determine the dependency of EGFR degradation on lysosomal or proteasomal pathways, EGF treatments were performed in the presence of leupeptin or bortezomib. Importantly, neither compound showed any cytotoxic effects (Fig. 7C and D). Blocking the proteasomal pathway with bortezomib prior to the addition of EGF inhibited EGFR degradation in HEV-infected cells, while uninfected cells still displayed a significant decrease in EGFR levels (Fig. 7E and F). Conversely, treatment with leupeptin, inhibiting lysosomal degradation, showed no decrease in EGFR levels in uninfected cells, but a reduction was evident in persistently HEV-infected cells (Fig. 7H and I). As a control for these treatments, levels of p62 were examined (Fig. 7G and J). Interestingly, HEV-replicating cells treated with leupeptin displayed no increase in p62 protein amount, while the respective uninfected control showed a significant induction.

To summarize, EGFR degradation sets on at an earlier time point post activation with EGF in persistently HEV-infected cells. Additionally, EGFR degradation in HEV-replicating cells appears to be dependent on the proteasomal pathway to a large extent, while the impact of the lysosomal system is less pronounced.

### Downstream signaling of EGFR is delayed in persistently HEV-infected cells

**Fig 8:**
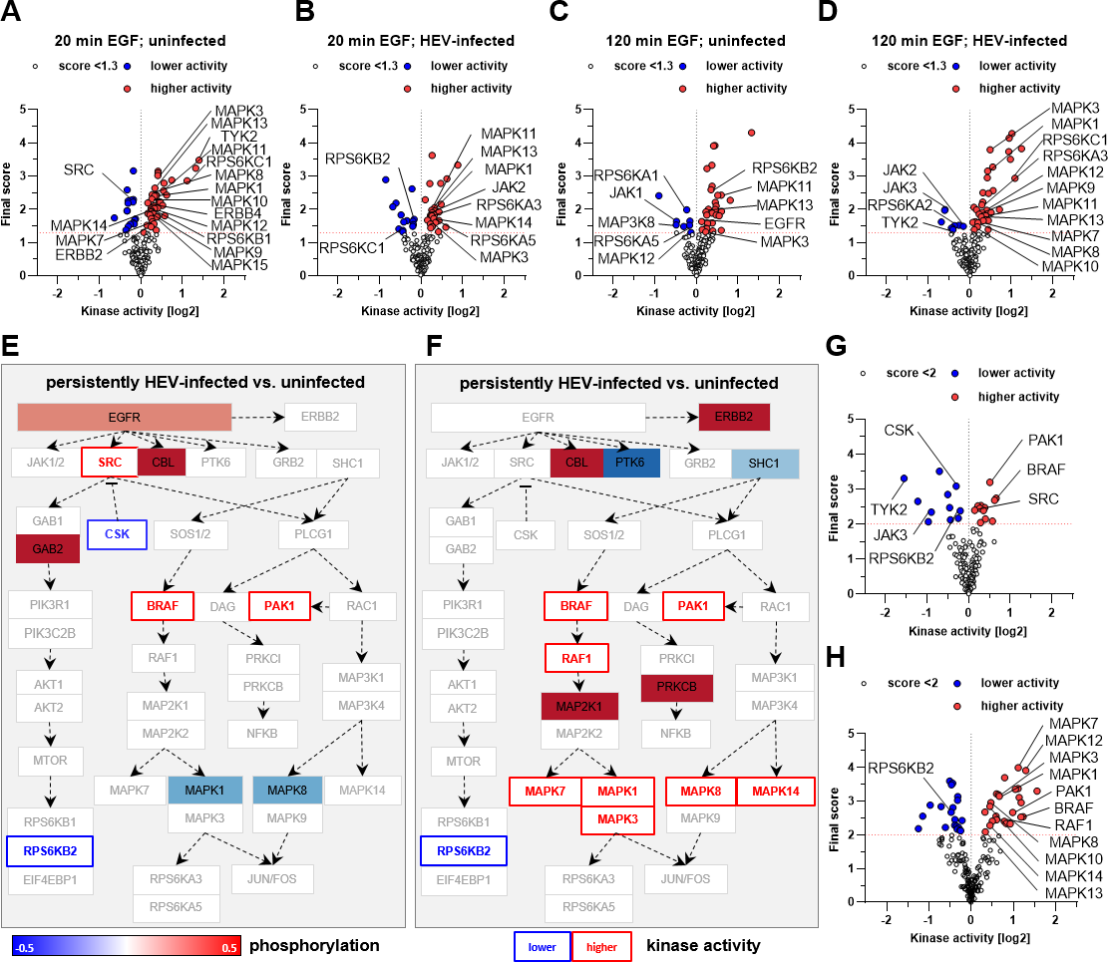
EGFR activation and signaling are delayed in persistently HEV-infected cells. (**A–D**) Volcano plot for predicted kinase activity. Shown are significantly up- (red dots) or downregulated (blue dots) kinases after EGF (50 ng/mL) treatment as compared with the respective untreated control. Shown are uninfected (**A**) and persistently HEV-infected (**B**) cells upon 20 min EGF treatment or uninfected (**C**) and persistently HEV-infected (**D**) cells upon 120 min EGF treatment. A mean final score above 1.3 was considered to be significant. (**E and F**) Change in peptide-phosphorylation and kinase activity in EGFR-mediated signaling cascades in persistently HEV-infected compared with uninfected A549 cells upon EGF (50 ng/mL) treatment for 20 min (**E**) or 120 min (**F**). Fill color represents extent of differential phosphorylation with <0 meaning lower and >0 meaning higher phosphorylation in log2 space; cut-off for depiction: *P* < 0.05. Coloring of font represents the absolute differential kinase activity with <0 meaning lower and >0 meaning higher activity in log2 space; cut-off for depicted kinases: final score > 2. Pathway maps are based on rearranged template from WikiPathways [EGFR-signaling (WP437)]. (**G and H**) Volcano plot for predicted kinase activity. Shown are significantly up- (red dots) or downregulated (blue dots) kinases in persistently HEV-infected compared with uninfected A549 cells upon EGF (50 ng/mL) treatment for 20 min (**G**) or 120 min (**H**). A mean final score above 2 was considered to be significant. Kinases in EGFR-mediated signaling cascades that were significantly changed in their activity were labeled by name.

Given that HEV modulates key regulatory processes of EGFR signaling, such as receptor internalization and degradation, we investigated the impact of HEV on EGFR downstream signaling. Comprehensive kinome analyses were conducted, comparing phosphorylation levels and kinase activities of multiple EGFR downstream targets in EGF-treated uninfected or HEV-infected cells as compared with their respective untreated control (Fig. 8).

Investigation of the EGFR-mediated signaling cascade regarding kinase activity after EGF treatment revealed a full downstream activation within 20 min after stimulation in uninfected cells, while it was less pronounced for HEV-replicating cells (Fig. 8A and B). Interestingly, HEV-replicating cells exhibited complete activation of downstream kinases after 2 h of stimulation, while signaling in uninfected cells began to cease (Fig. 8C and D). Comparison of EGF stimulated persistently HEV-infected and uninfected cells regarding peptide phosphorylation and kinase activity of EGFR-mediated signaling targets confirmed the above-described findings. Heightened phosphorylation and activity for initial kinases in the signaling pathway within 20 min stimulation were observed in HEV-infected cells as compared with uninfected cells (Fig. 8E and G), while full downstream activation was displayed after 2 h of stimulation in HEV-replicating cells (Fig. 8F and H).

These findings indicate a delayed EGFR-mediated signal transduction in cells persistently infected with HEV.

### Viral replication and spread are favored by silencing and inhibition of EGFR

The observed decrease in EGFR protein amount, in combination with an HEV-dependent EGFR-mediated signaling delay, raised the question whether EGFR silencing or impairment of its functionality could affect the HEV life cycle.

**Fig 9:**
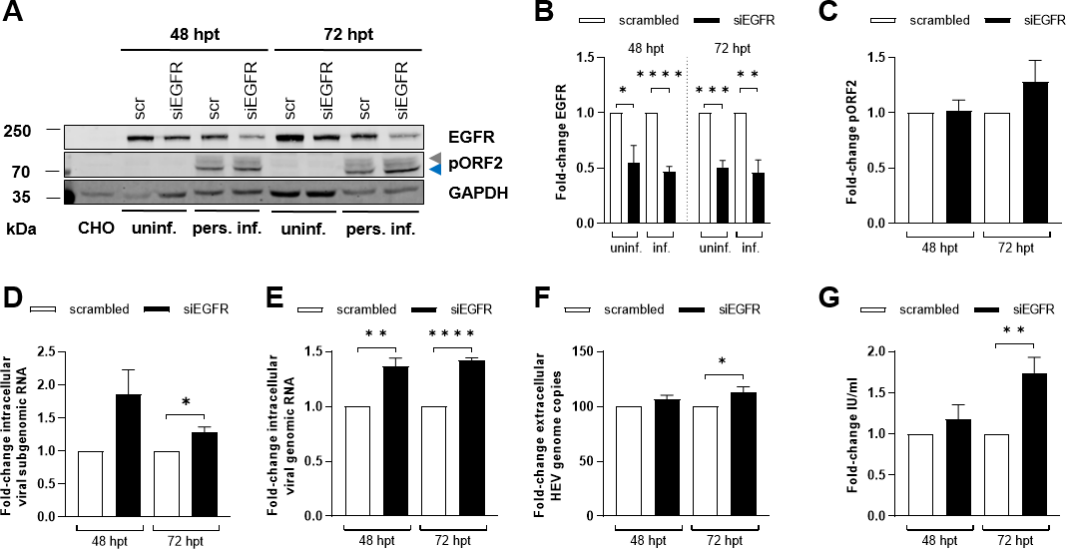
EGFR silencing favors viral replication and spread. (**A**) Representative western blot of EGFR and HEV pORF2 (blue = unglycosylated, gray = glycosylated) in lysates of uninfected and persistently HEV-infected A549 cells 48 h or 72 h post transfection (hpt) with siEGFR or scrambled control (scr). GAPDH was detected as loading control. Epithelial chinese hamster ovary (CHO) cell lysate was loaded as control, lacking EGFR expression. (**B and C**) Quantification of the relative amount of EGFR (**B**) and HEV pORF2 (**C**) in panel A. Values are normalized to the respective GAPDH amount. Statistics refer to scrambled-transfected cells; *N* = 4. (**D and E**) Relative change in the amount of intracellular viral subgenomic (**D**) and genomic (**E**) RNA measured by qRT-PCR in persistently HEV-infected A549 cells after transient transfection with siRNA for 48 h and 72 h. Values are normalized toward the respective amount of reference gene hRPL27. Statistics refer to scrambled-transfected cells; *N* = 3. (**F**) Fold change of extracellular HEV RNA from persistently HEV-infected A549 cells determined by qRT-PCR. Statistics refer to scrambled-transfected cells; *N* = 7. (**G**) Viral release of siRNA-transfected, persistently HEV-infected A549 cells shown as infectious units per mL (IU/mL) determined in an end-point dilution assay (TCID_50_). Values were compared with scrambled-transfected cells; *N* = 3. Statistical analyses were performed using unpaired *t*-test: *P < 0.05, **P < 0.01, ***P < 0.001, and ****P < 0.0001.

**Fig 10:**
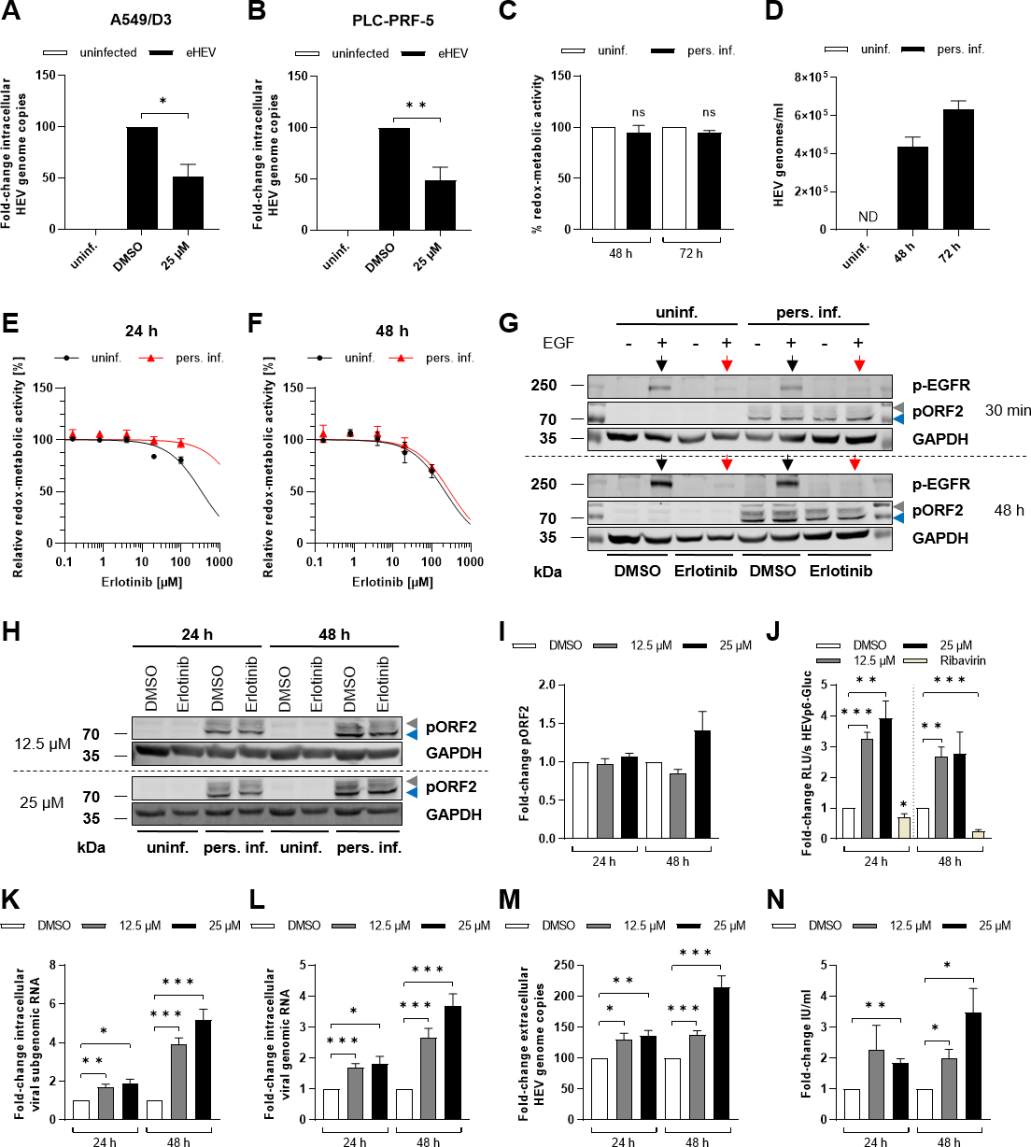
Inhibition of EGFR signaling favors viral replication and spread. (A and B) Relative change in intracellular HEV genome copies of A549/D3 (A) or PLC-PRF-5 cells (B) after 1 h infection with eHEV in the presence of 25 µM erlotinib assessed by HEV entry assay; *N* = 3. (C) Relative metabolic activity of uninfected and persistently HEV-infected A549 cells 48 h or 72 h post seeding assessed by a PrestoBlue cell viability assay; *N* = 3. (D) Amount of extracellular HEV RNA from persistently HEV-infected A549 cells 48 h or 72 h post seeding determined by qRT-PCR; *N* = 8. ND: not detectable. (E and F) Relative metabolic activity of uninfected and persistently HEV-infected A549 cells treated with erlotinib (0.16 µM–100 µM) for 24 h (E) or 48 h (F) assessed by a PrestoBlue cell viability assay; *N* = 3. (G) Representative western blot of phosphorylated EGFR (p-EGFR), HEV pORF2, and GAPDH in lysates of A549/D3 (uninf.) and persistently HEV-infected A549 cells stimulated with EGF (50 ng/mL) for 30 min in presence (red arrow) or absence (black arrow) of erlotinib (12.5 µM). Cells were pre-treated with erlotinib for 30 min or 48 h. (H) Representative western blot of HEV pORF2 and GAPDH in lysates of uninfected and persistently HEV-infected A549 cells treated with 12.5 µM or 25 µM erlotinib. (Ι) Quantification of the relative amount of HEV pORF2 in panel Η; *N* = 3. Values are normalized to the respective GAPDH amount. (J) Erlotinib-based effects on viral genome replication determined via subgenomic luciferase reporter assay (HEV genotype 3 subgenomic replicon) shown as fold change in relative light units per second (RLU/s) of Gaussia luciferase. Ribavirin (100 µM) was used as control. (K and L) Relative change in amount of intracellular viral subgenomic (K) and genomic (L) RNA after erlotinib treatment (12.5 µM or 25 µM) for 24 h and 48 h determined by qRT-PCR; *N* ≥ 4. Values are normalized toward the respective amount of reference gene hRPL27. (M) Fold change of extracellular HEV RNA from erlotinib-treated persistently HEV-infected A549 cells determined by qRT-PCR; *N* ≥ 5. (N) Viral release of erlotinib-treated persistently HEV-infected A549 cells shown as infectious units per mL (IU/mL) determined by TCID_50_. All statistics refer to untreated control (DMSO). Unpaired *t*-test: ns: not significant; *P < 0.05, **P < 0.01, and ***P < 0.001.

EGFR silencing was assessed through western blot in both uninfected and HEV-infected cells, revealing a significant reduction by half upon silencing as compared with the respective control (Fig. 9A and B). While intracellular HEV pORF2 protein levels showed only a tendency for an increase upon EGFR silencing (Fig. 9C), intracellular viral subgenomic and genomic transcripts were significantly increased 72 h post EGFR knockdown as compared with the control (Fig. 9D and E). Furthermore, quantification of extracellular HEV genomes and viral titers were in line with intracellular results, showing a significant increase 72 h post knockdown (Fig. 9F and G). In comparison to siRNA-based EGFR silencing, which is limited by the transfection efficiency, drug-based methods targeting receptor functionality offer the advantage of reaching all cells uniformly and achieving effective results. Therefore, untreated and HEV-replicating cells were treated with the well-known EGFR inhibitor erlotinib to explore potential effects on key steps of the viral life cycle. As previously demonstrated in other studies, erlotinib caused a significant reduction in viral entry in our lung-derived A549 subclone cell culture model, as well as in the liver-derived Alexander cell line PLC-PRF-5 (Fig. 10A and B). The extent to which this antiviral effect on viral entry is crucial for an established HEV infection was assessed by treatment of persistently HEV-infected A549 cells with 25 µM erlotinib (Fig. 10C and D). Under these conditions, erlotinib efficiently inhibited EGFR phosphorylation after EGF treatment at both 30 min and 48 h without any cytotoxic effect (Fig. 10E through G). Interestingly, while erlotinib treatment exhibited inhibitory effects on HEV entry via EGFR inhibition, no significant change in intracellular HEV pORF2 protein levels, as analyzed by western blot, was observed (Fig. 10H and I). In fact, there was even a tendency indicating a potential increase. In accordance with this finding, the EGFR inhibition via erlotinib resulted in significantly enhanced viral replication, which was associated with higher intracellular viral subgenomic and genomic RNA levels (Fig. 10J through L). Similarly, higher extracellular HEV RNA levels and viral titers were observed as a result of erlotinib-mediated EGFR inhibition (Fig. 10M and N).

**Fig 11:**
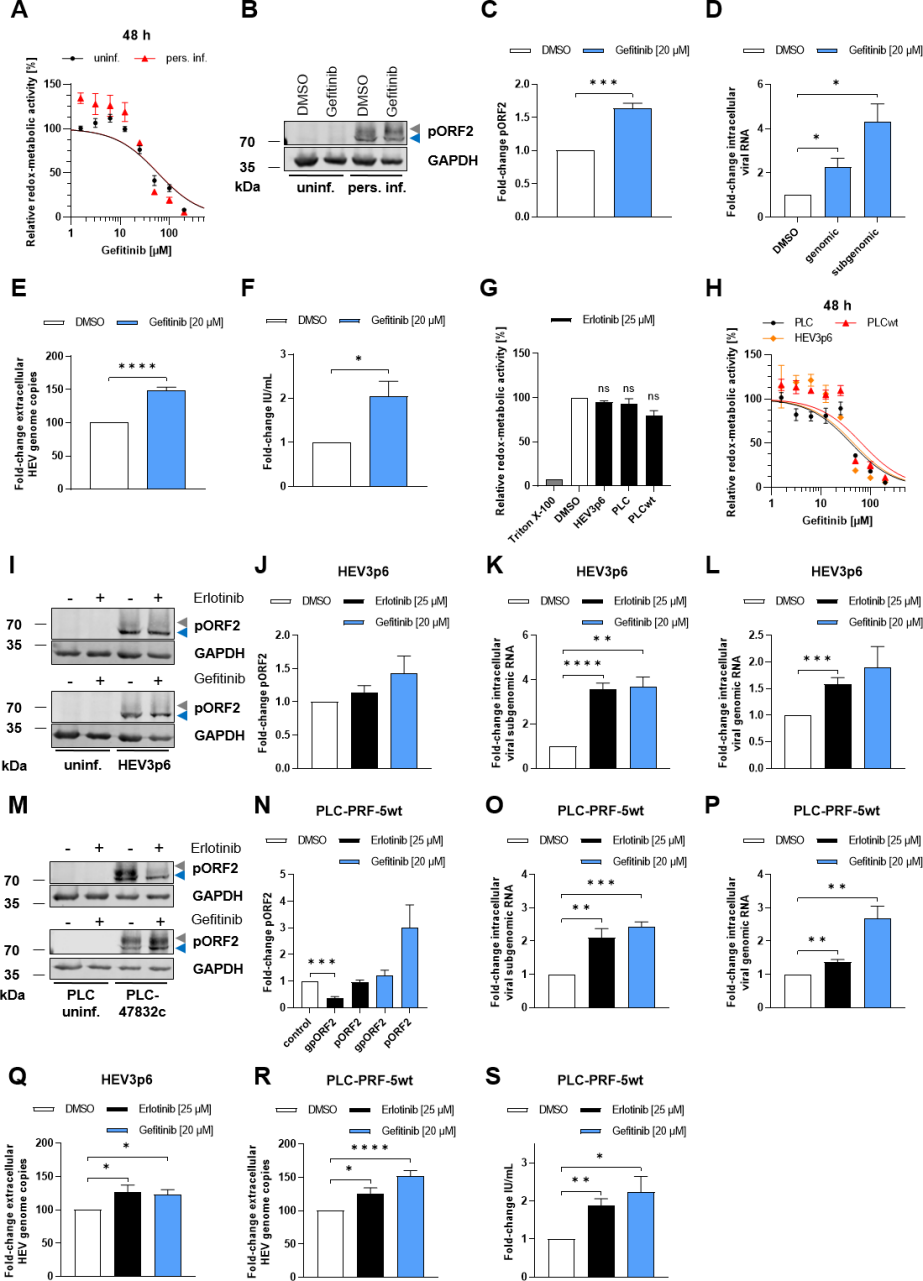
Gefitinib and erlotinib promote the HEV viral life cycle in both lung-derived A549 and liver-derived PLC-PRF-5 cells. (**A**) Relative metabolic activity of uninfected and persistently HEV-infected A549 cells treated with gefitinib (1.56 µM–200 µM) for 48 h assessed by a PrestoBlue cell viability assay; *N* = 3. (**B**) Representative western blot of HEV pORF2 and GAPDH in lysates of uninfected and persistently HEV-infected A549 cells treated with 20 µM gefitinib. (**C**) Quantification of the relative amount of HEV pORF2 in panel B; *N* = 3. Values are normalized to the respective GAPDH amount. (**D**) Relative change in amount of intracellular viral subgenomic and genomic RNA after gefitinib treatment (20 µM) for 48 h determined by qRT-PCR; *N* = 3. Values are normalized toward the respective amount of reference gene hRPL27. (**E**) Fold change of extracellular HEV RNA from 48 h gefitinib-treated (20 µM) persistently HEV-infected A549 cells determined by qRT-PCR; *N* = 6. (**F**) Viral release of gefitinib-treated (20 µM) persistently HEV-infected A549 cells shown as infectious units per mL (IU/mL) determined by TCID_50_. All statistics refer to untreated control (DMSO). (**G**) Relative metabolic activity was assessed using PrestoBlue cell viability assay in persistently HEV3 KernowC1 p6-infected A549/D3 (HEV3p6), uninfected PLC-PRF-5 (PLC), and persistently HEV3 47832c-infected PLC-PRF-5 (PLCwt) cells treated with 25 µM erlotinib for 48 h; *N* = 3. (**H**) Relative metabolic activity of HEV3p6, PLC, and PLCwt cells treated with gefitinib (1.56 µM–200 µM) for 48 h; *N* = 3. (**I**) Representative western blot of HEV pORF2 and GAPDH in lysates of uninfected and HEV3p6 cells treated with 25 µM erlotinib or 20 µM gefitinib for 48 h. (**J**) Quantification of the relative amount of HEV pORF2 in panel I; *N* = 3. Values are normalized to the respective GAPDH amount. (**K and L**) Relative change in amount of intracellular viral subgenomic (**K**) and genomic (**L**) RNA of HEV3p6 cells after treatment with 25 µM erlotinib or 20 µM gefitinib for 48 h; *N* ≥ 3. Values are normalized toward the respective amount of reference gene hRPL27. (**M**) Representative western blot of HEV pORF2 and GAPDH in lysates of uninfected and persistently HEV3 47832c-infected PLC-PRF-5 cells treated with 25 µM erlotinib or 20 µM gefitinib for 48 h. (**N**) Quantification of the relative amount of HEV pORF2 (gpORF2 = glycosylated pORF2; pORF2 = unglycosylated pORF2) in panel M; *N* = 3. Values are normalized to the respective GAPDH amount. (**O and P**) Relative change in amount of intracellular viral subgenomic (**O**) and genomic (**P**) RNA of persistently HEV3 47832c-infected PLC-PRF-5 cells after treatment with 25 µM erlotinib or 20 µM gefitinib for 48 h; *N* ≥ 4. Values are normalized toward the respective amount of reference gene hRPL27. (**Q and R**) Fold change of extracellular HEV RNA from HEV3p6 (**Q**) and persistently HEV3 47832c-infected PLC-PRF-5 cells (**R**) after treatment with 25 µM erlotinib or 20 µM gefitinib for 48 h; *N* ≥ 4. (**S**) Viral release of 25 µM erlotinib or 20 µM gefitinib-treated persistently HEV3 47832c-infected PLC-PRF-5 cells shown as infectious units per mL (IU/mL) determined by TCID_50_; *N* ≥ 3. All statistics refer to untreated control (DMSO). Unpaired *t*-test: ns: not significant; *P < 0.05, **P < 0.01, ***P < 0.001, and ****P < 0.0001.

The aforementioned pro-viral effects of EGFR inhibition on HEV were validated by another EGFR inhibitor, gefitinib, where an increase in viral load was observed both intracellularly and extracellularly (Fig. 11A through F). Here, the increase in intracellular and extracellular HEV RNA (Fig. 11D and E) was associated with an increase in viral pORF2 protein amount (Fig. 11B and C). Especially noteworthy is the validation of the aforementioned pro-viral effects of EGFR inhibition using both inhibitors, not only with another HEV strain, HEV3 KernowC1 p6 (Fig. 11G through L and Q), but also in another cell culture system, the liver-derived PLC-PRF-5 cells (Fig. 11M through P, R and S).

In conclusion, both EGFR silencing and inhibition exert a pro-viral effect, with erlotinib leading to an increased replication of HEV in persistently infected cells. Importantly, these data indicate that the inhibitory effect of EGFR inhibition on HEV entry is overcompensated by its proviral effect on later steps of HEV life cycle.

### Intracellular cholesterol homeostasis disrupted by EGFR inhibition

**Fig 12:**
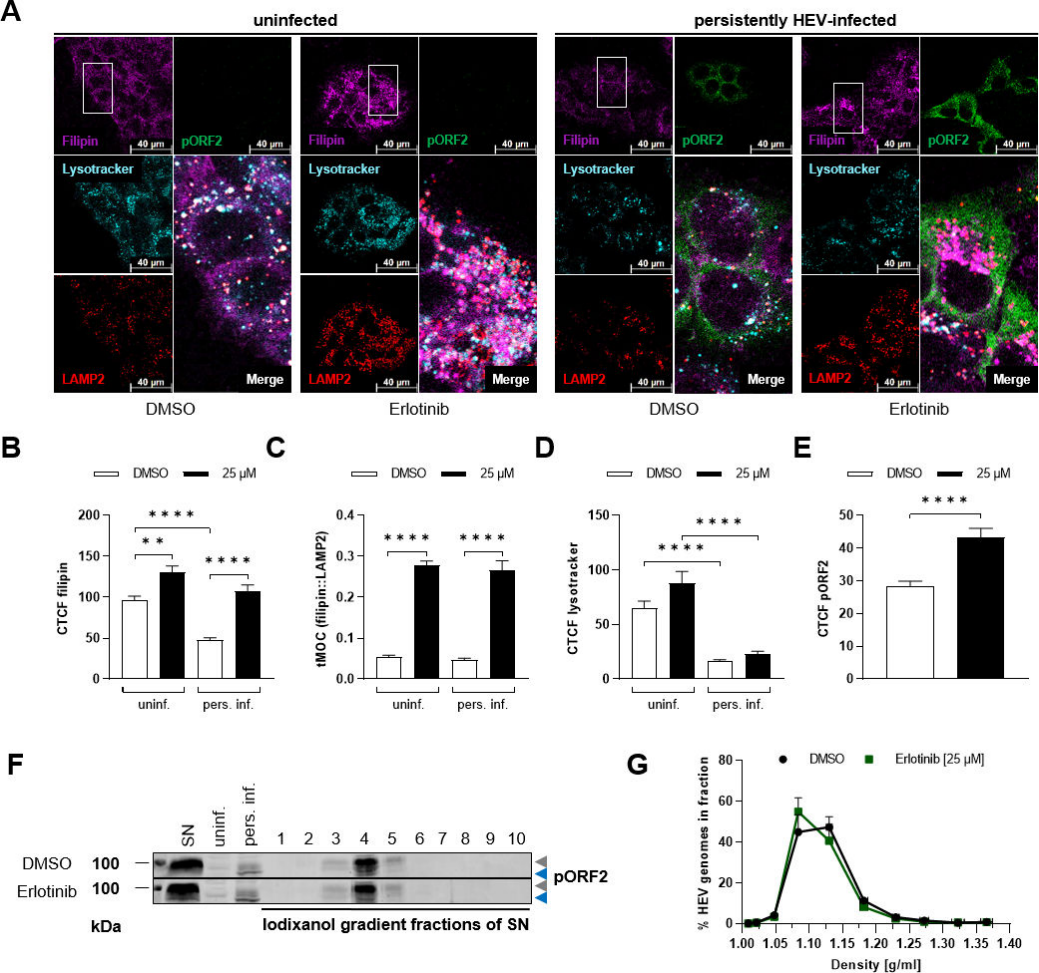
Erlotinib treatment interferes with intracellular cholesterol homeostasis. Uninfected and persistently HEV-infected A549 cells were treated with erlotinib (25 µM) for 48 h and fixed with FA. (**A**) Representative confocal microscopy images immunostained for HEV pORF2 (green) and LAMP2 (red). Intracellular cholesterol was counterstained with filipin (magenta) and acidic organelles with lysotracker (cyan). Cell shown within the zoomed merge section is indicated within the filipin image. Scale bar: 40 µm. (**B**) Quantification of filipin CTCF per cell in panel A; *n* ≥ 19. Microscopy was performed on Leica TCS SP8 system with 100× objective (numerical aperture 1.4). (**C**) Quantification of colocalization (tMOC) per cell for magenta filipin signal present in red LAMP2 areas in panel A; *n* ≥ 20. (**D**) Quantification of lysotracker intensity as CTCF per cell in panel A; *n* ≥ 15. (**E**) Quantification of HEV pORF2 intensity as CTCF per cell in panel A; *n* ≥ 23. (**F**) Representative western blot of HEV pORF2 (blue = unglycosylated, gray = glycosylated) in lysates of uninfected and persistently HEV-infected A549 cells as well as density-gradient fractions loaded with supernatant (SN) from persistently HEV-infected A549 cells. (**G**) Extracellular HEV RNA in fractions of density-gradients as determined by qRT-PCR; depicted as % of whole genomes in gradient; *N* = 5. All statistics refer to respective untreated control (DMSO) or uninfected A549 cells as indicated. Unpaired *t*-tests with Holm-Sidak correction for multiple-group comparison: **P < 0.01 and ****P < 0.0001.

A recent study established a connection between intracellular cholesterol levels and the life cycle of HEV, revealing that higher intracellular cholesterol levels favored HEV degradation in the lysosomal compartment, whereas lower intracellular cholesterol levels led to increased release of HEV progeny (25). In light of the proviral effects triggered by EGFR inhibition, it was investigated if this correlates with a change in the intracellular cholesterol amount. Therefore, immunofluorescence microscopy analysis of intracellular cholesterol via filipin staining was performed. In addition, the lysosomal compartment was monitored with LAMP2-specific staining and lysotracker (Fig. 12A).

Here, erlotinib-induced increase in both uninfected and persistently HEV-infected cells was observed (Fig. 12B). Notably, untreated persistently HEV-infected cells exhibited inherently lower filipin signals as compared with the respective uninfected cells. The intracellular cholesterol was accumulated in lysosomal structures as evidenced by filipin stain co-localization with LAMP2-positive structures in erlotinib-treated cells. This was independent of HEV infection (Fig. 12C). Moreover, lysotracker signal indicated a significant reduction in lysosomal acidification in persistently HEV-infected cells as compared with the uninfected control (Fig. 12D). Consistent with the observed pro-viral effects of erlotinib on HEV, pORF2 signal intensity showed a significant increase following EGFR inhibition (Fig. 12E). While the western blot analysis only indicated an upward trend in pORF2 levels after erlotinib treatment, the effect becomes more pronounced and reaches statistical significance when analyzed at the single-cell level.

As HEV can be released as quasi-enveloped particles, it was investigated whether the erlotinib-induced lysosomal accumulation of intracellular cholesterol affected particle density of released HEV virions. For this purpose, density gradient centrifugation was performed, revealing no differences in viral particle density upon EGFR inhibition (Fig. 12F and G).

Taken together, these data indicate that EGFR inhibition leads to the accumulation of intracellular cholesterol in LAMP2-positive structures. While lysosomal acidification is only slightly affected by erlotinib, significantly less lysotracker signal is detected in persistently HEV-infected cells. The accumulation and impaired acidification suggest dysfunctional lysosomes, which might impair lysosomal mobilization of esterified cholesterol. Thus, storage of cholesterol in lysosomes leads to a decrease on the intracellular cholesterol level outside of this compartment. In accordance to this, an increase of HEV pORF2 protein amount can be detected upon EGFR inhibition.

### Induction of autophagosomal structures by EGFR inhibition aiding HEV release

**Fig 13:**
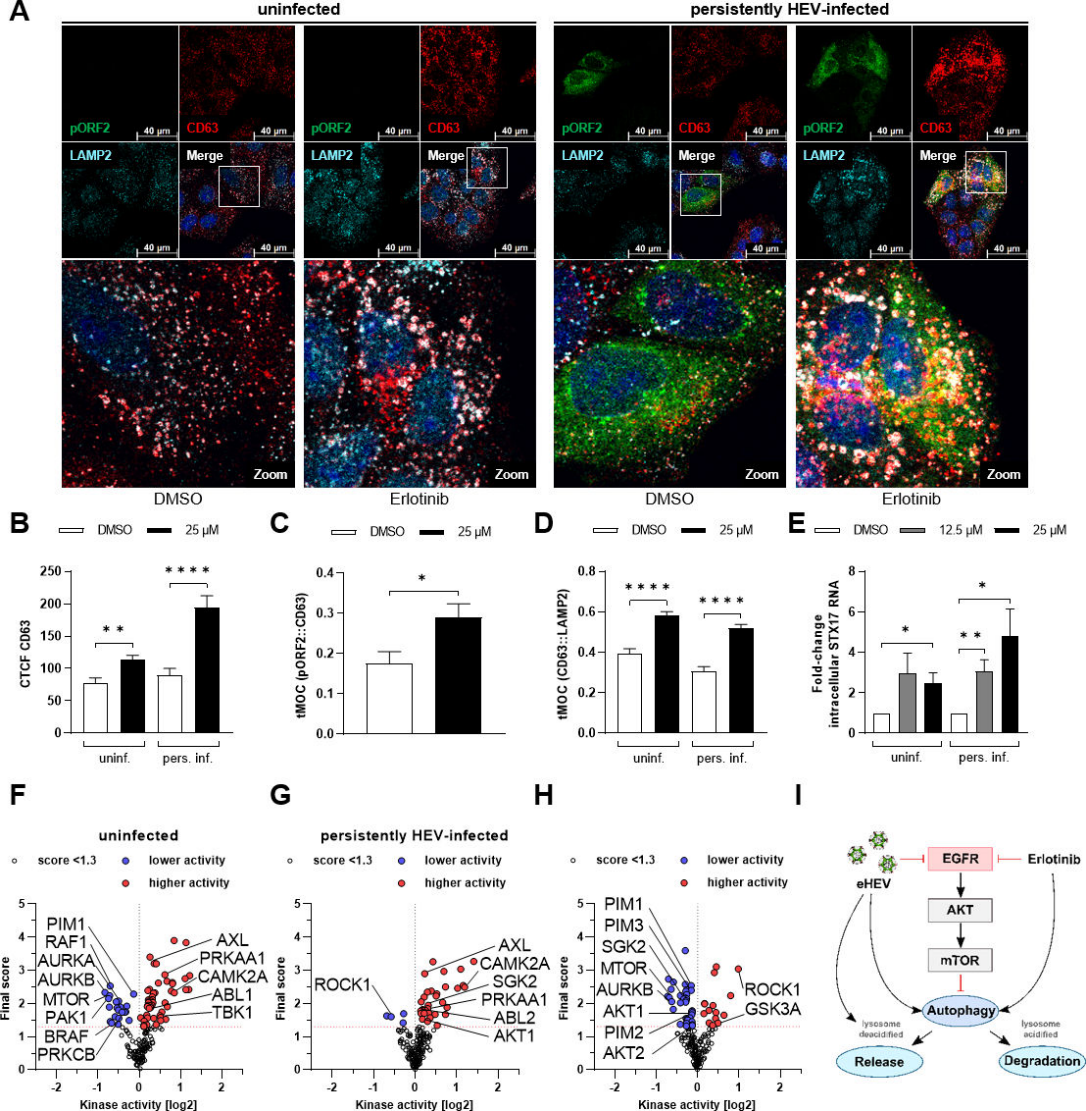
EGFR inhibition promotes the endosomal system used by HEV for viral release. Uninfected and persistently HEV-infected A549 cells were treated with erlotinib (25 µM) for 48 h and fixed with EtOH. (**A**) Representative confocal microscopy images immunostained for HEV pORF2 (green), CD63 (red), and LAMP2 (cyan). Nuclei were counterstained with DAPI (blue). Cell shown in the zoom section is indicated within the merge image. Scale bar: 40 µm. Microscopy was performed on Leica TCS SP8 system with 100× objective (numerical aperture 1.4). (**B**) Quantification of CD63 CTCF per cell in panel A; *n* ≥ 17. (**C**) Quantification of colocalization (tMOC) per cell for green HEV pORF2 signal present in red CD63 areas in panel A; *n* ≥ 14. (**D**) Quantification of colocalization (tMOC) per cell for red CD63 signal present in cyan LAMP2 areas in panel A for uninfected and persistently HEV-infected cells; *n* ≥ 10. (**E**) Relative change in amount of intracellular syntaxin 17 (STX17) mRNA after erlotinib treatment (12.5 µM or 25 µM) for 48 h in uninfected and persistently HEV-infected A549 cells. Values refer to untreated control and were normalized toward the respective reference gene hRPL27; *N* = 5. All statistical analyses were performed using unpaired *t*-test related to respective untreated A549 cells (DMSO): *P < 0.05, **P < 0.01, and ****P < 0.0001. (**F–H**) Volcano plot for predicted kinase activity. Shown are significantly up- (red dots) or downregulated (blue dots) kinases either upon 48 h erlotinib treatment (25 µM) in uninfected (**F**) and persistently HEV-infected (**G**) A549 cells compared to the untreated control (DMSO) or upon HEV infection comparing untreated persistently HEV-infected A549 cells with respective uninfected control (**H**). A mean final score above 1.3 was considered to be significant. Several autophagy-related kinases that were significantly changed in their activity were labeled by name. (**I**) Schematic representation of HEV- and erlotinib-based induction of the autophagy pathway. HEV impairment of lysosomal acidification changes the fate of autophagosomal structures.

Aforementioned data clearly indicate that both HEV infection and erlotinib treatment affect the endolysosomal compartment. HEV utilizes late endosomal structures, particularly MVBs, for virion formation and release. Given the observed pro-viral effect of erlotinib on viral replication and extracellular viral titers, its influence on MVBs was explored using immunofluorescence microscopy. Cluster of differentiation 63 (CD63), an MVB marker, and LAMP2 were co-stained with HEV pORF2 (Fig. 13A).

Here, an induction of CD63 upon EGFR inhibition was observed, which was independent from HEV infection (Fig. 13B). However, persistently HEV-infected cells displayed a more pronounced increase in CD63 level as compared with uninfected cells. Interestingly, the co-localization between pORF2 and CD63 was significantly increased upon erlotinib treatment (Fig. 13C). Furthermore, erlotinib treatment increased the co-localization between CD63- and LAMP2-positive structures (Fig. 13D) and induced the expression of the soluble N-ethylmaleimide-sensitive-factor attachment receptor (SNARE) protein syntaxin 17 (Stx17) (Fig. 13E). These observations indicates toward an erlotinib-dependent promotion of autophagosomal structures. To validate this observation, kinome analyses were conducted comparing kinase activities of erlotinib-treated persistently HEV-infected and uninfected cells with their respective untreated controls. Erlotinib-induced changes in the kinome profile revealed an upregulation of several autophagy-related kinases for uninfected (Fig. 13F) and HEV-infected cells (Fig. 13G). Interestingly, persistent HEV infection itself resulted in a kinome profile inducing autophagy-related pathways (Fig. 13H). This is in accordance with the relevance of autophagy-related processes for MVB-dependent release in the form of exosomes.

These findings highlight a dependency between the late endosomal system and EGFR functionality. Thus, EGFR inhibition by erlotinib upregulates the autophagosomal system and promotes subcellular localization of pORF2 within these structures (summarized within Fig. 13I).

### Reduced innate immune response upon EGFR inhibition

Given the role of EGFR in viral defense and the observed pro-viral effect associated with erlotinib treatment, we examined the expression of interferon-stimulated genes (ISGs). Cells were treated with erlotinib, and ISG expression was analyzed using qRT-PCR.

**Fig 14:**
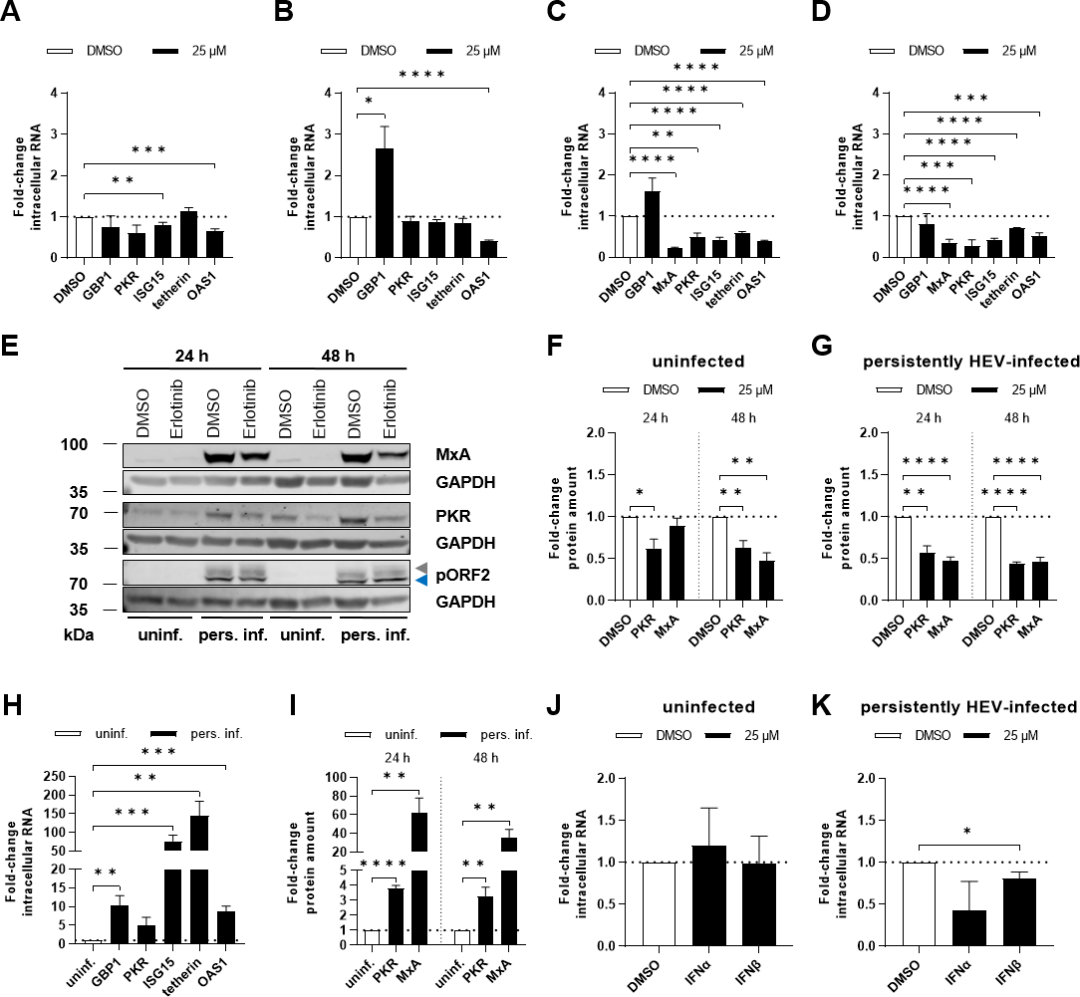
Erlotinib treatment reduces the host’s innate immune response. Uninfected and persistently HEV-infected A549 cells were treated with erlotinib (25 µM) for 24 h and 48 h. (**A and B**) Relative change in expression of ISGs measured via qRT-PCR in uninfected A549 cells after 24 h (**A**) or 48 h (**B**) erlotinib treatment. Values were referred to untreated control (DMSO) after normalization toward the respective amount of reference gene hRPL27; *N* ≥ 4. (**C and D**) Relative change in expression of ISGs measured via qRT-PCR in persistently HEV-infected A549 cells after 24 h (**C**) or 48 h (**D**) erlotinib treatment. Values referred to untreated control (DMSO) after normalization toward the respective amount of reference gene hRPL27; *N* ≥ 4. (**E**) Representative western blot of MxA, PKR, and HEV pORF2 (blue = unglycosylated, gray = glycosylated) in lysates of uninfected and persistently HEV-infected A549 cells treated with erlotinib for 24 h and 48 h. GAPDH was detected as loading control. (**F and G**) Quantification of the relative amount of ISGs in panel E for uninfected (**F**) and persistently HEV-infected (**G**) A549 cells. Values refer to respective untreated control (DMSO) after normalization toward the respective GAPDH amount as loading control; *N* = 4. (**H**) Relative change in expression of ISGs measured via qRT-PCR in untreated (48 h) persistently HEV-infected A549 cells as compared with the respective uninfected control. Values were normalized toward the respective amount of reference gene hRPL27. Controls were set to a value of 1; *N* ≥ 5. (**I**) Quantification of the relative amount of ISGs in panel E for untreated (DMSO) persistently HEV-infected A549 cells referred to respective uninfected control; *N* = 4. (**J and K**) Relative change in expression of interferon-alpha (IFNα) and beta (IFNβ) measured via qRT-PCR in uninfected A549 cells (**J**) or persistently HEV-infected A549 cells (**K**) after 48 h of 25 µM erlotinib treatment. Values were referred to untreated control (DMSO) after normalization toward the respective amount of reference gene hRPL27; *N* ≥ 5. Unpaired *t*-test: *P < 0.05, **P < 0.01, ***P < 0.001, and ****P < 0.0001.

EGFR inhibition led to a significant reduction in the expression of interferon-stimulated gene 15 (ISG15) and oligoadenylate synthetase 1 (OAS1) after 24 h of erlotinib treatment in uninfected cells (Fig. 14A). After 48 h of erlotinib treatment, only OAS1 expression levels were diminished (Fig. 14B). Notably, in HEV-replicating cells, several ISGs were downregulated after 24 h (Fig. 14C) and 48 h of treatment (Fig. 14D). Western blot analyses and subsequent quantification of the ISG human myxovirus resistance protein 1 (MxA) and protein kinase R (PKR) yielded similar results, showing decreased protein amounts for both MxA and PKR after erlotinib treatment in persistently HEV-infected cells and uninfected control cells (Fig. 14E through G). However, there is the tendency for a more pronounced decrease in HEV-infected cells. It has to be stated that relative values are shown. The basal expression of the analyzed ISGs is in HEV-infected cells significantly higher as compared with the negative control cells (Fig. 14H and I). The reduction in ISG levels correlated with a decreasing trend of the levels of interferon alpha- and beta-specific transcripts after erlotinib treatment. While this effect was absent in uninfected cells, it was apparent in persistently HEV-infected cells, which had previously displayed more pronounced effects on ISGs (Fig. 14J and K).

In summary, EGFR inhibition strongly impairs innate immune response, as reflected by a decrease in ISG expression.

## DISCUSSION

The EGFR serves as a pivotal regulator of cellular homeostasis by orchestrating downstream signaling cascades that influence cell proliferation, differentiation, and survival. Its involvement in the pathogenesis of various viruses, including HBV, HCV, ZIKV, IAV, adenovirus, and HCMV, underlines its diverse roles in host-cell entry, viral replication, and modulation of host-defense responses (35–37, 39, 59, 60). Recent insights have extended its significance to early stages of HEV infection, particularly in playing a supportive role within virion entry (42). However, the results of this study provoke a more differentiated role for EGFR within the HEV life cycle. Its role is dependent on the stage of HEV infection, as we discovered a reduced EGFR amount and impaired function in persistently HEV-infected cells, as discussed in the subsequent sections.

Persistent HEV infection significantly reduces EGFR expression and protein level across two different HEV genotype 3 strains. Besides the infected cells, adjacent cells exposed to the infectious conditions also exhibit slight changes in EGFR levels. These cells could potentially be infected but are not yet showing HEV replication. However, the specificity of the observed effect is corroborated by the observation that cure of HEV-positive cells by ribavirin reverses the effects of HEV infection on EGFR. Loss of HEV replication is associated with the reversal of the HEV EGFR level and distribution as observed for the uninfected control cells. Despite increased receptor internalization into lysosomal structures during infection, there is no discernible impact on EGFR half-life. Our data evidence that the reduction in EGFR levels mainly results from transcriptional downregulation upon infection rather than increased lysosomal degradation. Interestingly, concomitant with the observed HEV-dependent reduction in EGFR amount, there is a difference between uninfected and persistently HEV-infected cells regarding EGFR regulation upon activation. Accelerated EGFR internalization in HEV-replicating cells indicates HEV-dependent heightened EGFR flux or reduced receptor recycling capacity due to the diminished EGFR pool upon infection. Moreover, as EGFR degradation begins early after stimulation of the receptor in persistently HEV-infected cells, EGFR levels are prematurely depleted, contributing to the scarcity of the EGFR recycling pool and capacity in HEV-infected cells. Furthermore, we demonstrated that this EGFR degradation after stimulation does not solely depend on lysosomal degradation but predominantly relies on proteasomal-driven degradation upon HEV infection. It is known that initial steps of EGFR degradation are proteasome dependent, with subsequent steps relying on the lysosomal system (61–64). Here, we show that HEV differentially regulates this proteasomal EGFR depletion and lysosomal receptor downmodulation.

Our findings indicate in multiple lines of evidence that this differential regulation is based on HEV infection disturbing lysosomal functionality. Given that HEV infection interfere with lysosomal acidification, an impairment of lysosomal functionality is likely. In line with this, blocking lysosomal functionality has no significant effect on p62 protein levels and EGFR degradation upon EGF stimulation relies more on proteasomal degradation than lysosomal pathways in HEV-replicating cells. A dysfunctional lysosomal degradation pathway in HEV-infected cells aligns with the dynamics of the HEV viral life cycle, where viral release depends on quasi-envelopment within late endosomal structures prioritizing exosomal pathways over lysosomal degradation. Thus, inhibiting lysosomal acidification shifts the cellular balance toward release, promoting HEV egress. Notably, the disruption of lysosomal pH with bafilomycin A1 increases HEV virus particle release as previously reported (17). The described mechanism of impairing the lysosomal compartment to facilitate viral egress parallels recent findings in the context of SARS-CoV2 infection (65, 66). Concordantly, this HEV-dependent interference with lysosomal functionality might also affect cholesterol homeostasis in the endosomal system, which HEV heavily relies on. Since lysosomal cholesterol esterase activity depends on an acidic environment, lysosomal dysfunctionality is accompanied with impaired mobilization of cholesterol, thus leading to a reduced cellular cholesterol level outside of lysosomes, favoring HEV replication and release (25).

Due to the beforementioned HEV-dependent changes in EGFR regulation, affecting its signal initiation and termination, a significant delay in EGFR-regulated signaling is in place for HEV. This delay may create an optimal environment for the establishment of viral persistence. The delayed response to extracellular stimuli not only influences the host’s response to the pathogen but also ensures isolation of the host cell. Similar mechanisms have been described for viruses like HCMV or human adenovirus (33, 35, 59). The manipulation of EGFR expression and the associated downregulation of its functionality plays a critical role in persistently HEV-infected cells. On the one hand, this is highlighted by EGFR silencing and on the other hand by inhibition with EGFR tyrosine kinase inhibitors (TKIs) like erlotinib and gefitinib, as both display pro-viral effects on several steps within the HEV life cycle enhancing intracellular and extracellular viral load. While TKI-induced EGFR internalization, as previously described, may contribute to the observed reduction in viral entry, its proviral effects on viral replication and release during an established infection counteract this effect (67–69). As a result, the use of erlotinib as an antiviral agent against advanced HEV infection is not recommended, emphasizing that viral entry is not the sole determinant affecting viral replication and life cycle.

Interestingly, inhibition of EGFR by erlotinib causes an accumulation of intracellular cholesterol in lysosomal structures. As discussed before, this disturbance of the cellular cholesterol homeostasis might favor HEV release and replication (25). In addition to the decrease in extralysosomal cholesterol levels, we observe a significant impact of erlotinib treatment on the autophagosomal system. By downregulating autophagy-related kinase activities such as mammalian target of rapamycin (mTOR), proviral integration Moloney virus kinase (PIM) and proto-oncogene, serine/threonine kinase (Raf1), or induction of the autophagy-related SNARE protein STX17 expression, formation of autophagosomal structures is promoted (70–74). Notably, erlotinib-based relative induction of autophagy is less pronounced in persistently HEV-infected cells, as HEV infection alone induces autophagy through inhibition of key kinases such as mTOR, protein kinase B (Akt1/2), and PIM1-3, which is consistent with previous publications (75). In particular, the structural protein pORF3 of HEV genotype 1 was found to induce cellular autophagy (76). The induction of autophagosomal structures, along with the previously discussed impaired lysosomal acidification and functionality upon HEV infection, promotes the pathway for viral release. Considering that MVBs are the compartments exploited by HEV for exosomal release, the observed localization of the viral capsid protein pORF2 in MVBs upon EGFR inhibition strongly indicates an enhanced viral release. Furthermore, the autophagosomal system is directly linked to HEV replication, as inhibition of autophagy-related pathways results in a decreased HEV genotype 3 replication (75). Interestingly, emerging evidence suggests a potential role for MVBs in HEV replication, given that the viral pORF1 polyprotein, responsible for viral replication, predominantly localizes in this compartment (52). In light of this, the induction of autophagy and MVBs by inhibiting EGFR with erlotinib supports and increases viral replication. This aligns with our observations, highlighting the interconnected role of autophagy in both HEV replication and the promotion of viral release.

While recognizing the notable significance of the endosomal system in the viral life cycle of HEV, our investigation shifts to another vital aspect affecting viral infections—the innate immune response. Originally recognized for its role in cell growth and development, EGFR is increasingly acknowledged as a key player in the intricate network regulating the innate immune response. The suppression of EGFR signaling by erlotinib reveals a notable decrease in several ISGs linked to antiviral responses. Our findings indicate that erlotinib may already compromise interferon expression, thereby contributing to the observed phenotype. Moreover, interferon type I-dependent signaling via Janus kinases (JAK) and signal transducers and activators of transcription proteins (STAT) has been found to depend on Raf1 (77). Thus, inhibiting Raf1 can effectively suppress the innate immune response. Kinome analyses have uncovered a downregulation of Raf1 kinase activity following erlotinib treatment, providing support for the observed effects of erlotinib on ISGs. The observation of suppressed innate immunity upon EGFR inhibition is particularly interesting considering the identified interference with EGFR signaling in the context of HEV infection. By disrupting EGFR signaling, an environment conducive to viral infection is established, shedding light on a critical aspect of HEV infection: the infection-associated disturbance of innate immunity and the potential involvement of EGFR in this process.

**Fig 15:**
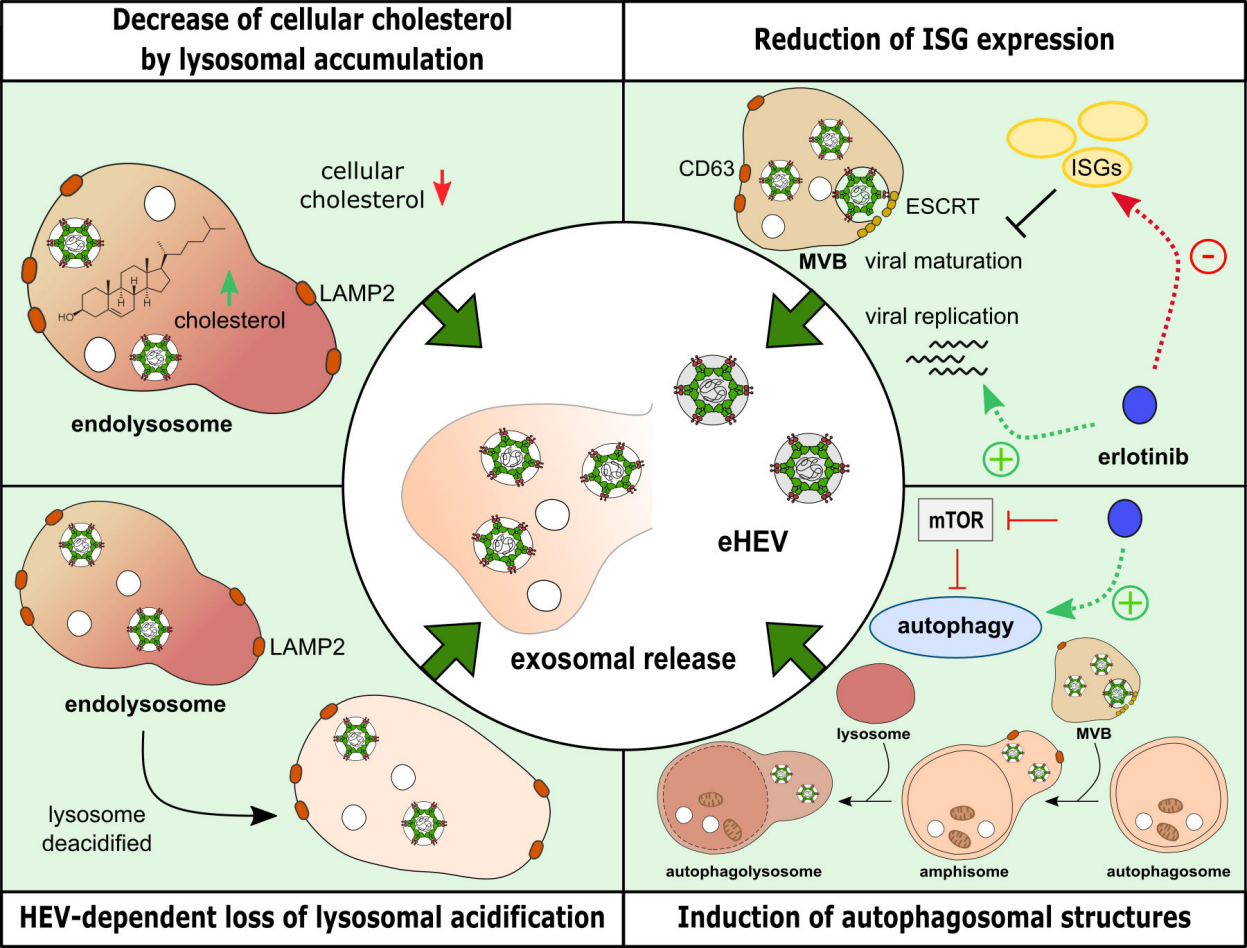
Deciphering the complex puzzle: erlotinib’s pro-viral impact is based on an intricate molecular interplay. The pro-viral influence of erlotinib treatment through EGFR inhibition seems to exhibit greater complexity. Erlotinib-induced accumulation of cholesterol in lysosomal structures exerts a pro-viral effect on HEV by simultaneously reducing cellular cholesterol level. Moreover, the induction of autophagosomal structures and the decrease in ISG expression upon erlotinib treatment, coupled with HEV-induced impairment of lysosomal acidification, aid this effect. This underlines a highly regulated interplay that ultimately culminates in the pro-viral impact of erlotinib.

**Fig 16:**
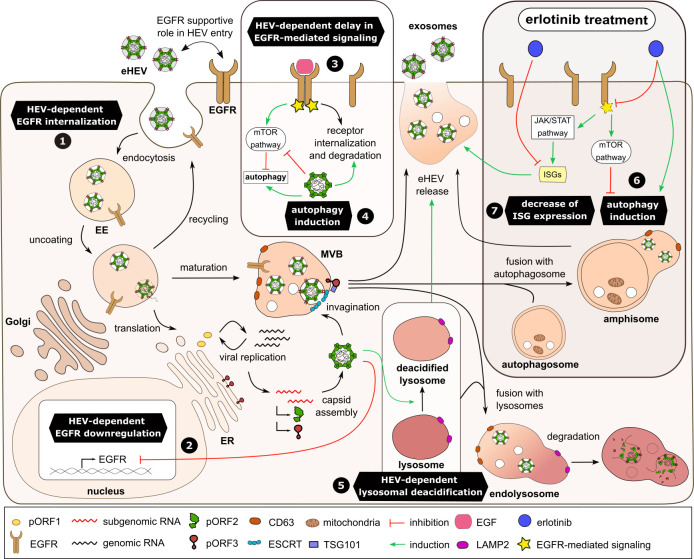
EGFRs role in the HEV life cycle. Persistently HEV-infected cells display an enhanced EGFR internalization (1), alongside with downregulation of EGFR expression (2). Activation of the EGFR with EGF induces early receptor internalization and degradation in HEV-replicating cells, causing a significant delay in EGFR-related signaling (3). HEV infection inhibits autophagy-related kinases (mTOR pathway), leading to the formation of autophagosomal structures for viral replication and release (4). An HEV-dependent interference with the lysosomal degradation pathway by inhibiting lysosomal acidification promotes exosomal egress (5). Inhibiting EGFR with erlotinib increases viral replication, both extracellular and intracellular viral transcripts, and release of infectious particles. This pro-viral impact is attributed to erlotinib-induced activation of the autophagosomal system (6) and impaired expression of ISGs (7).

**Fig 17:**
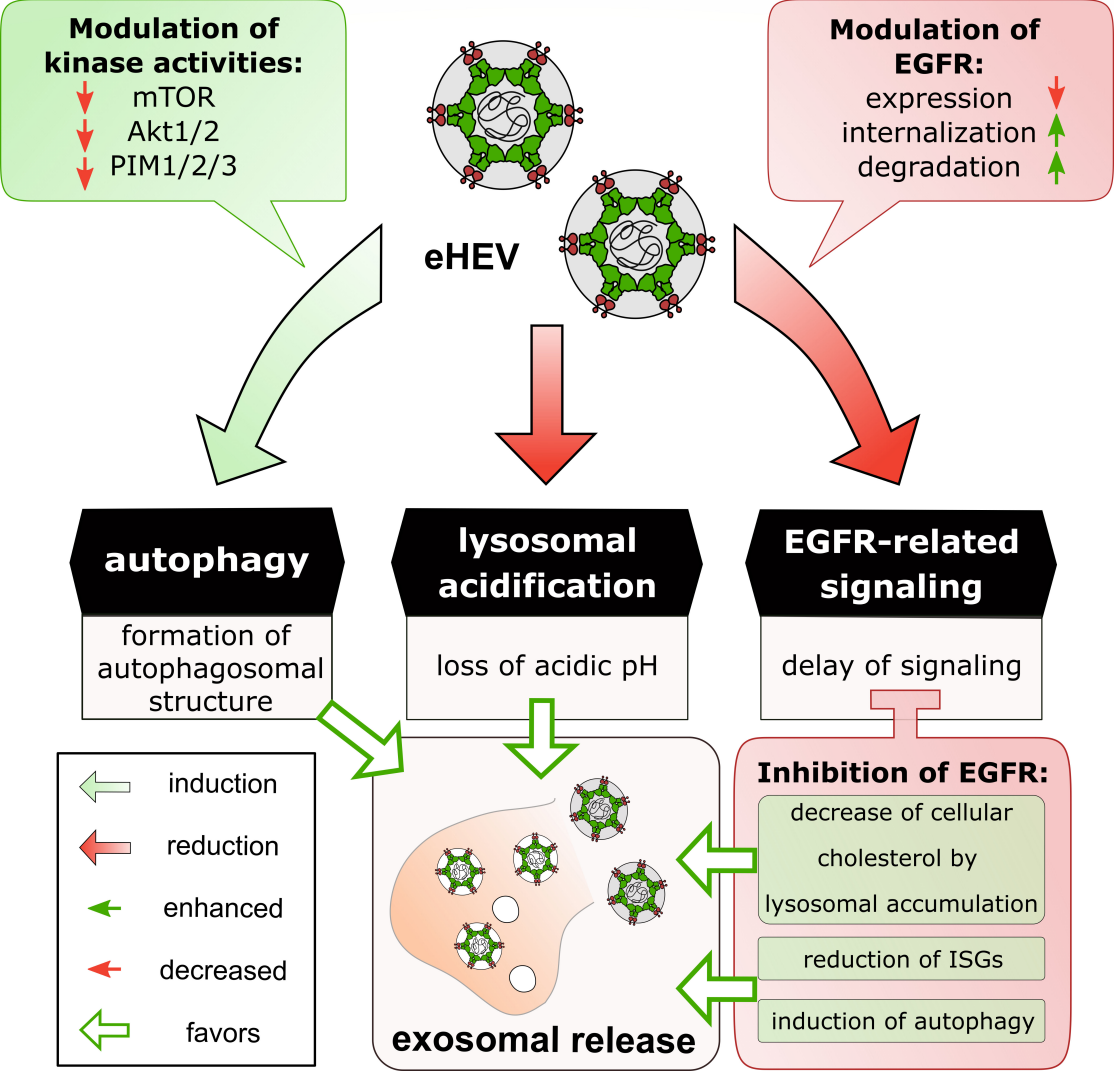
Schematic representation summarizing the EGFR-HEV crosstalk.

In summary, we dissect HEV-EGFR-crosstalk in HEV-infected cells and the tightly regulated multifaceted pro-viral impact of EGFR inhibition on the HEV life cycle. The pro-viral effect of EGFR inhibition depends in part on decreased expression of ISGs, induction of autophagosomal structures, and impaired mobilization of cholesterol esters in lysosomes, as depicted in Fig. 15. We identify EGFR as a novel host factor for HEV, demonstrating that infection compromises EGFR-regulated pathways, thereby promoting viral replication, spread, and evasion from the innate immune response, as illustrated in Fig. 16 and 17. Additionally, we propose, for the first time, an HEV-dependent interference with lysosomal acidification as a novel mechanism for orchestrating viral release during infection.

## Data Availability

All relevant study data can be made available upon request.
